# Application of In Silico QSAR and Molecular Docking Studies to a Series of Xanthine-Based Analogues and Design, Synthesis and Pharmacological Evaluation of Identified New Potential Selective MAO-B Inhibitors

**DOI:** 10.3390/ph19060892

**Published:** 2026-06-04

**Authors:** Yavor Mitkov, Emilio Mateev, Iva Valkova, Stefan Kostov, Magdalena Kondeva-Burdina, Alexander Zlatkov

**Affiliations:** 1 Department of Pharmaceutical Chemistry, Faculty of Pharmacy, Medical University–Sofia, 2 Dunav str., 1000 Sofia, Bulgaria; y.mitkov@pharmfac.mu-sofia.bg (Y.M.); e.mateev@pharmfac.mu-sofia.bg (E.M.); stefankostov01@gmail.com (S.K.); 2 Department Chemistry, Faculty of Pharmacy, Medical University–Sofia, 2 Dunav str., 1000 Sofia, Bulgaria; ivalkova@pharmfac.mu-sofia.bg; 3 Department of Pharmacology, Pharmacotherapy and Toxicology, Faculty of Pharmacy, Medical University–Sofia, 2 Dunav str., 1000 Sofia, Bulgaria; mkondeva@pharmfac.mu-sofia.bg

**Keywords:** caffeine derivatives, neuroprotection, QSAR, molecular docking, MAO-A/B

## Abstract

**Background/Objectives**: Methylxanthines, such as caffeine, exhibit neuroprotective properties in neurodegenerative conditions, partly linked to modulation of monoamine oxidase B (MAO-B) and oxidative stress pathways. This work aimed to design, synthesize and functionally characterize new caffeine-8-methylthioglycolic acid derivatives as selective MAO-B inhibitors with neuroprotective potential. **Methods**: A QSAR model was built on 94 studies of xanthine derivatives to guide the design of ten new semi- and thiosemicarbazides (**Jas1**–**Jas10**), followed by molecular docking to human MAO-B (PDB: **2V5Z**) using Glide, GOLD and MM-GBSA binding free energy calculations. The target compounds were synthesized in relatively high yields, structurally confirmed by spectroscopic methods and tested in vitro for *h*MAO-A/B inhibition, as well as for neurotoxicity and neuroprotection in isolated mouse brain synaptosomes, mitochondria and microsomes under 6-hydroxydopamine (6-OHDA), *tert*-butyl hydroperoxide (*t*-BuOOH) and Fe/ascorbate (Fe^2+^/AA)-induced oxidative stress. **Results**: Docking and MM-GBSA identified **Jas6** and **Jas7** as the most stable MAO-B binders, with binding free energies approaching those of safinamide. All derivatives inhibited *h*MAO-A and *h*MAO-B in the submicromolar range, with **Jas2** and **Jas6** showing the highest MAO-B selectivity indices. At 100 µM, the series produced mild but significant pro-oxidant and cytotoxic effects when applied alone, yet under oxidative stress all compounds, especially **Jas2** and **Jas6**, markedly preserved synaptosomal and mitochondrial viability, maintained glutathione levels, and reduced malondialdehyde production. **Conclusions**: The caffeine-based semi- and thiosemicarbazides, particularly **Jas2** and **Jas6**, emerge as promising selective MAO-B inhibitors with pronounced antioxidant and neuroprotective activity, supporting their further optimization as multitarget candidates for neurodegenerative disorders such as Parkinson’s disease.

## 1. Introduction

Methylxanthines are a class of bioactive compounds widely recognized for their pharmacological effects as central nervous system stimulants, bronchodilators, coronary vasodilators, and diuretics. In addition to these well-established activities, increasing evidence highlights their relevance in neurodegenerative, cardiovascular, metabolic, and reproductive disorders. However, their therapeutic use is limited by potential toxicity and adverse effects associated with excessive intake, necessitating the development of optimized derivatives with improved safety and efficacy profiles [1]. Recent studies further support the pharmacological relevance of xanthine-based scaffolds, particularly in relation to oxidative stress modulation, mitochondrial protection, and enzyme regulation [2,3,4,5].

The biological activity of methylxanthines is closely associated with the purinergic signaling system, a fundamental regulatory network involved in neurotransmission, vascular regulation, and energy metabolism across multiple tissues, including the central nervous system, pancreas, and cardiovascular system. This system comprises nucleotides, nucleosides, metabolizing enzymes, and purinergic receptors, all of which contribute to maintaining cellular homeostasis. Dysregulation of purinergic signaling has been implicated in pathological conditions such as insulin resistance, vascular injury, inflammation, and neurodegeneration. Importantly, excessive purinergic activation contributes to oxidative stress and cellular damage during hypoxic and inflammatory conditions. For instance, caffeine has been shown to modulate purinergic signaling under hypoxia, thereby attenuating inflammatory and oxidative damage induced by elevated extracellular ATP and reactive nitrogen species [6].

From a pharmacological perspective, monoamine oxidase B (MAO-B) represents a validated therapeutic target in Parkinson’s disease (PD), as its overactivity contributes to dopamine degradation and increased production of reactive oxygen species, thereby exacerbating oxidative stress and neurodegeneration [7]. Consequently, MAO-B inhibitors are clinically established agents in PD management, providing both symptomatic relief and neuroprotective effects [8]. In this context, the development of novel MAO-B inhibitors with additional antioxidant properties remains a key strategy in neurodegenerative drug discovery [9].

Caffeine and its metabolites have been widely investigated for their neuroprotective potential in PD and other neurodegenerative disorders. The primary metabolite paraxanthine has demonstrated significant neuroprotective effects in neuronal models, independent of classical adenosine receptor antagonism or cAMP signaling pathways. Instead, its activity has been linked to modulation of intracellular calcium homeostasis via ryanodine receptor activation, which may indirectly influence mitochondrial function and oxidative stress regulation. Similarly, caffeine has been associated with reduced risk of cognitive decline and neurodegeneration, effects attributed to both antioxidant activity and modulation of adenosine receptor signaling pathways [10].

Xanthine derivatives constitute a well-established pharmacophore in medicinal chemistry and drug design, with structural modifications enabling fine-tuning of pharmacokinetic properties, receptor affinity, and enzyme inhibitory activity. Their versatility has led to extensive exploration as multifunctional scaffolds targeting oxidative stress-related enzymes, including monoamine oxidases (MAO-A and MAO-B), which are key contributors to reactive oxygen species (ROS) formation and neurodegenerative processes. In particular, MAO-B inhibition is strongly associated with neuroprotection in disorders such as Parkinson’s disease, as it reduces dopamine catabolism and limits oxidative-stress-mediated neuronal damage. Consequently, xanthine-based derivatives have attracted increasing interest as lead structures for the development of multifunctional neuroprotective agents combining MAO inhibition, antioxidant activity, and mitochondrial protection [7,11,12,13].

In this context, the present study reports the synthesis of a novel series of caffeine-derived semi- and thiosemicarbazides and evaluates their potential as multifunctional agents with combined antioxidant, neuroprotective, and monoamine oxidase inhibitory activities, supported by in silico modeling and in vitro biological assays.

## 2. Results

An in silico study including two approaches—QSAR methodology and molecular docking—was applied to organize data on previously observed structure–activity relationships and to propose a systematic series of structural modifications that lead to a new family of xanthines designated as selective MAOB inhibitors.

### 2.1. QSAR Model Build-Up

For the construction of an appropriate QSAR model, a dataset of 94 compounds reported in the literature was utilized [14,15,16,17,18]. Each compound structure was considered as composed of four fragments: the xanthine core, a heteroatom linked at the 8-position of the xanthine ring, a connecting bridge, and a terminal substituent opposite the xanthine core. Variations in these fragments were encoded using indicator variables that reflect the presence (1) or absence (0) of specific substituents (Table 1).

Descriptor selection via a genetic algorithm (GA) and QSAR model construction by multiple linear regression (MLR) was accomplished using the MDL QSAR v.2.0 software package. Statistical significance was assessed using the coefficient of determination (R^2^), standard error of estimation (SEE), and the Fisher criterion (F). The predictive ability of the models was evaluated by cross-validation and Y-scrambling tests.

As a result of the regression analysis (MLR), a linear structure–activity relationship was derived, described by the following equation:

pIC50 = 1.51 × C8O + 2.033 × C8S + 0.8341 × link(CH_2_)_4_ + 0.9699 × linkO − 1.681 × EndC_5_H_10_ − 0.994 × EndCH(CH_3_)_2_ + 0.5268 × End mBr benz + 0.7631 × End pBr benz + 0.4962 × End mCL benz + 0.6494 × End pCL benz + 4.64862, (1)

n = 94   r^2^ = 0.719   SEE = 0.544   F = 20.26   q^2^ = 0.638   r^2^scr = 0.120

**Figure 1:**
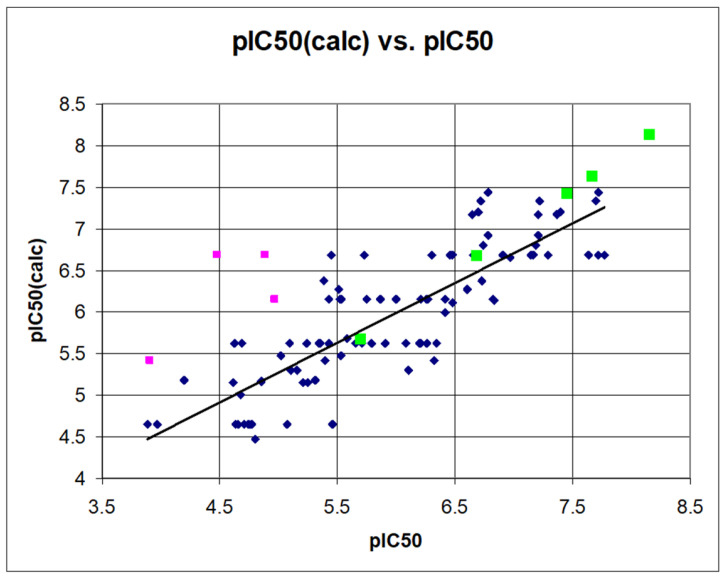
Correlation between experimentally determined (pIC_50_ from literature) and model-predicted MAO-B inhibitory activity for the studied compounds. Outlier compounds are indicated in pink. Model-predicted pIC_50_ values for new compounds are shown.

The correlation between experimentally determined and model-predicted MAO-B inhibitory activity (expressed as pIC_50_) for the studied compounds is shown in Figure 1.

**Figure 2:**
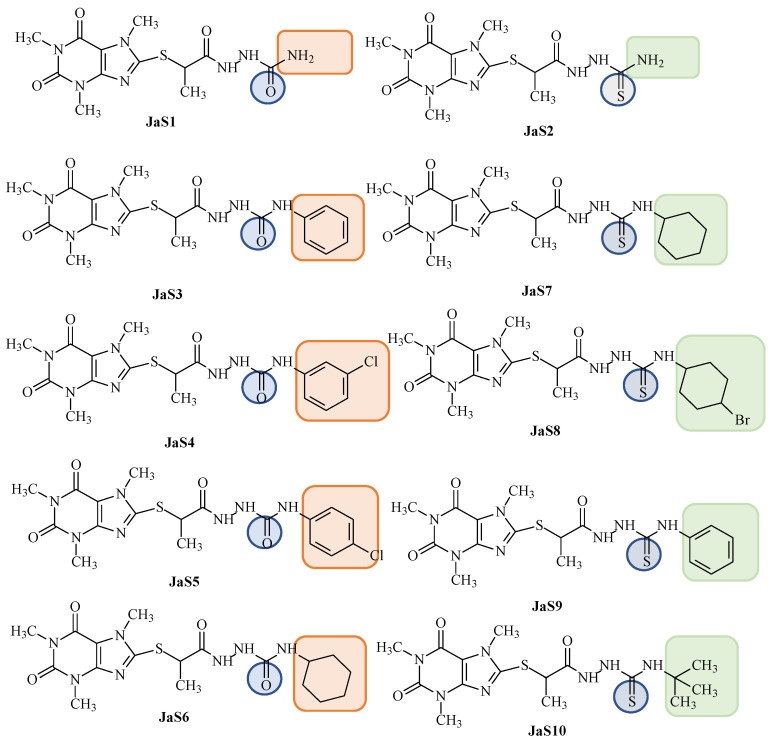
Xanthine-based compounds identified as potential MAO-B inhibitors.

Ten new compounds were designed (Figure 2), incorporating sulfur as the linking atom to the xanthine ring, a six-atom semicarbazide or thiosemicarbazide bridge, and terminal substituents previously included in the QSAR analysis.

The next section of this manuscript presents molecular docking studies for the ten newly designed compounds within the active site of MAO-B. These simulations aimed to evaluate the stability and binding interactions of the ligand–enzyme complexes, providing additional insights into their potential inhibitory effects. Following the computational analysis, all compounds were synthesized and their MAO-B inhibitory activities assessed experimentally in vitro. This integrated approach enabled validation of the predicted binding properties and offered a comprehensive evaluation of their effectiveness as inhibitors.

### 2.2. Molecular Docking

The xanthine-based compounds identified from the QSAR model presented in Figure 2 were subjected to molecular docking studies. The validation of the docking protocols was previously carried out through redocking, cross docking and ligand enrichment, which is reported in a recent study of our research group [19].

The designed compounds were initially docked into the active site of MAO-B (PDB ID: **2V5Z**) with the HTVS option in Glide (Maestro). Thereafter, the protocols were rescored with the more precise XP mode (Glide). To increase the robustness of the docking calculations, the docking program GOLD 5.3 was used for consensus docking. Moreover, the free binding energies of the complexes were calculated with MM/GBSA (Table 2).

To obtain more information about the active conformations of the docked xanthine-based ligands, all stable enzyme-ligand complexes were visualized and analyzed. The amino residues from the active site of MAO-B, which were involved in stabilization bonds, are provided in Supplementary Table S1.

**Figure 3:**
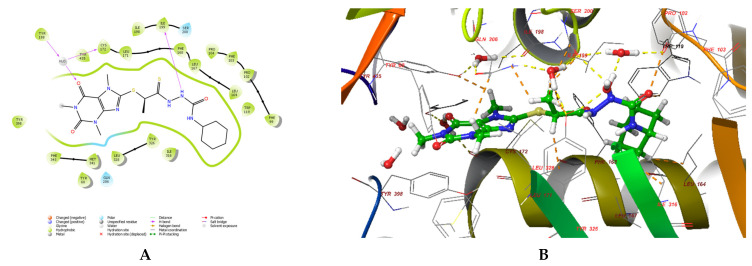
Major molecular interactions between **JaS7** and the active site of MAO-B (PDB: **2V5Z**) (**A**) in 2D form and (**B**) interactions in 3D form. The MAO-B enzyme is depicted in ribbons, the active amino acids are labeled in red, and the best ranked xanthine-based compound **JaS7** is colored by present elements.

Further visualizations of the active conformation and the stabilizing intermolecular forces between the most promising, according to the docking results, xanthine-based compound (**JaS7**) and the active site of MAO-B were conducted (Figure 3).

### 2.3. Synthesis

Based on our previous experimental results and those of the performed QSAR and molecular docking evaluations, we designed a small series of ten semi- and thiosemicarbazide caffeine derivatives with different potencies.

**Scheme 1:**
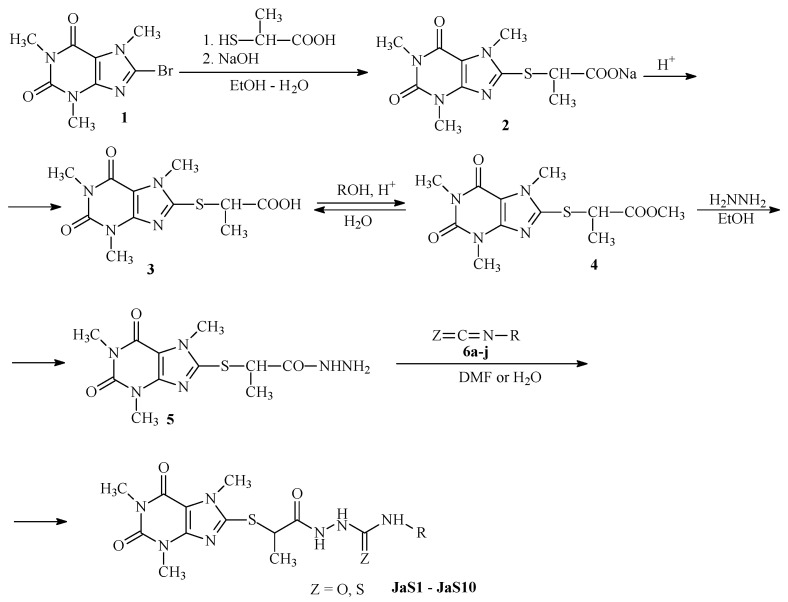
Overall synthetic scheme for the synthesis of the identified xanthine-based products.

The identified xanthine-based compounds were carefully evaluated for synthetic feasibility, and a total synthetic scheme was constructed (Scheme 1).

The hydrazide of caffeine 8-α-methylthioglycolic acid was previously synthesized by our research group [20]. The synthesis of the final semi- and thiosemicarbazides was performed using a molar ratio of reactants of 1:1.25, in either boiling water or dimethylformamide at 40 °C (Scheme 1). Reaction progress and completion were monitored by TLC, following the depletion of the hydrazide. The structures of the newly synthesized compounds were confirmed by IR, ^1^H and ^13^C NMR spectroscopy, and the corresponding characterization data are presented in the materials and methods section.

The results presented in Table 3 indicate that the reaction yields are significantly lower when conducted in an aqueous medium. The choice of solvent in the first two cases was based on the solubility of the reactants, with superior yields and shorter reaction times achieved in DMF.

Following successful synthesis, the compounds were subjected to in vitro evaluation to experimentally validate the in silico predictions.

### 2.4. Pharmacological In Vitro Evaluations

#### 2.4.1. Monoamine Oxidase A/B (MAO-A/B) Inhibitory Activity Assessment

Monoamine oxidase (MAO) activity for both recombinant human MAO-A (*h*MAOA) and MAO-B (*h*MAOB) was assessed using a fluorometric assay based on the Amplex^®^ UltraRed reagent and defined in Bautista-Aguilera et al. [21] and later adapted with minor changes by Kasabova-Angelova et al. [22].

**Figure 4:**
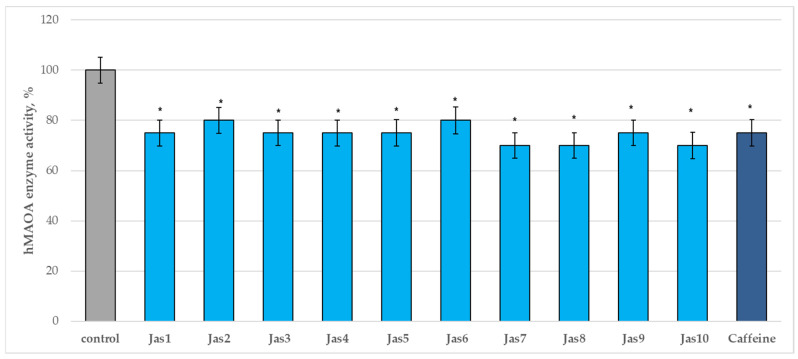
Effects of the **Jas1**–**Jas10** series and caffeine (at a concentration of 1 µM) on the activity of human recombinant MAO-A enzyme. * *p* < 0.05 compared to the control (pure *h*MAOA).

**Figure 5:**
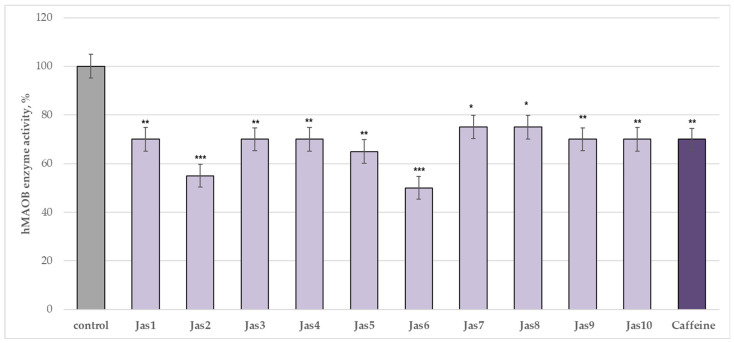
Effects of the **Jas1**–**Jas10** series and caffeine (at a concentration of 1 µM) on the activity of human recombinant MAO-B enzyme. * *p* < 0.05; ** *p* < 0.01; *** *p* < 0.001 compared to the control (pure *h*MAOB).

The **Jas1**–**Jas10** compounds all demonstrated significant inhibitory activity against human recombinant MAO-A and MAO-B enzymes. Specifically, **Jas1**, **Jas3**, **Jas4**, **Jas5**, **Jas9**, and caffeine inhibited *h*MAOA by 25%, **Jas2** and **Jas6** by 20%, and **Jas7**, **Jas8**, and **Jas10** by 30%, relative to the enzyme control (Figure 4). Regarding *h*MAOB inhibition, **Jas1**, **Jas3**, **Jas4**, **Jas9**, **Jas10**, and caffeine showed a 30% reduction; **Jas2** inhibited by 45%, **Jas6** by 50%, **Jas5** by 35%, and **Jas7** and **Jas8** by 25% (Figure 5).

The corresponding IC_50_ values were determined for all compounds to calculate their selectivity index (SI) towards *h*MAOB versus *h*MAOA (Table 4).

The results indicated that compounds **Jas2** and **Jas6** displayed the highest selectivity indices, indicating their promising potential as selective MAO-B inhibitors for further investigation.

#### 2.4.2. Effects on Isolated Mouse Brain Synaptosomes

**Figure 6:**
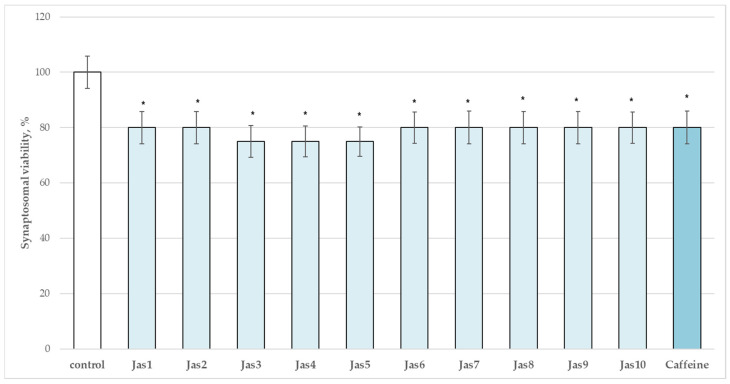
Effects of the **Jas1**–**Jas10** series and caffeine (at a concentration of 100 µM), applied individually, on synaptosomal viability. * *p* < 0.05 compared to the control (untreated synaptosomes).

**Figure 7:**
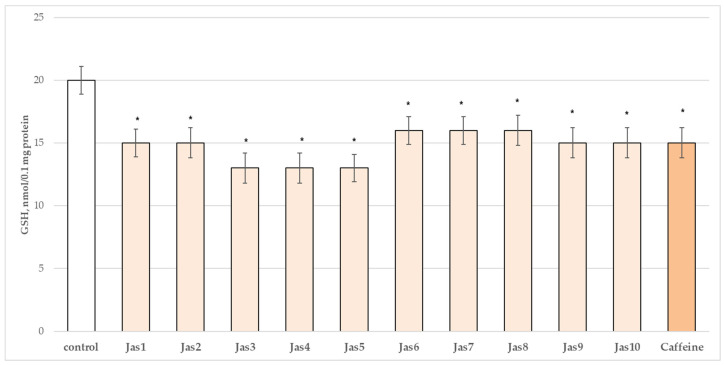
Effects of the **Jas1**–**Jas10** series and caffeine (at a concentration of 100 µM), applied individually, on glutathione (GSH) levels in isolated synaptosomes; * *p* < 0.05 compared to the control (untreated synaptosomes).

When applied individually at 100 µM to isolated mouse brain synaptosomes, all **Jas1**–**Jas10** compounds and caffeine, applied as a reference, exhibited a mild but statistically significant neurotoxic effect compared to untreated controls (Figure 6 and Figure 7). Synaptosomal viability was reduced by approximately 20–25% depending on the compound. The levels of reduced glutathione (GSH), a critical antioxidant marker, were also decreased in synaptosomes upon treatment with these compounds, with reductions ranging from 20 to 35% depending on the compound.

**Figure 8:**
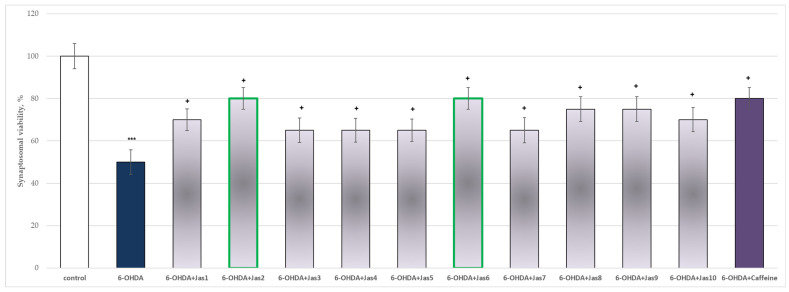
Effects of the **Jas1**–**Jas10** series and caffeine (at a concentration of 100 µM), under 6-OHDA-induced oxidative stress conditions, on synaptosomal viability. *** *p* < 0.001 compared to the control (untreated synaptosomes); + *p* < 0.05 compared to 6-OHDA-treated samples.

**Figure 9:**
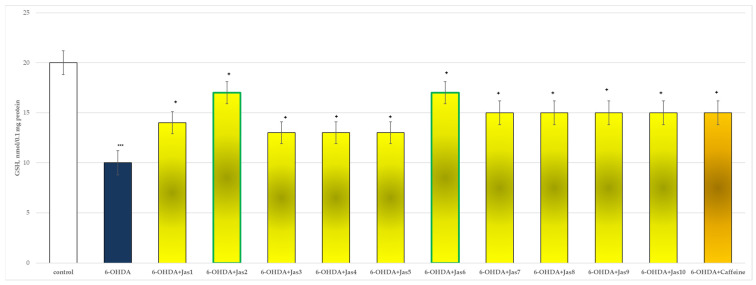
Effects of the **Jas1**–**Jas10** series and caffeine (at a concentration of 100 µM), under 6-OHDA-induced oxidative stress, on GSH levels in brain synaptosomes. *** *p* < 0.001 compared to the control (untreated synaptosomes); + *p* < 0.05 compared to 6-OHDA-treated samples.

Under oxidative stress conditions induced by 6-hydroxydopamine (6-OHDA, 150 µM), which mimics Parkinson’s disease-related neurotoxicity, all **Jas1**–**Jas10** compounds demonstrated significant neuroprotective effects by preserving synaptosomal viability (Figure 8) and maintaining GSH levels (Figure 9) compared to 6-OHDA treatment alone. Among these, **Jas2** and **Jas6** showed the most pronounced protective effects while exhibiting the lowest neurotoxicity when applied individually.

Under conditions of 6-OHDA-induced oxidative stress, **Jas1** preserved the GSH level by 40% in a statistically significant manner; **Jas2** and **Jas6** by 70%; **Jas3**, **Jas4**, and **Jas5** by 30%; and **Jas7**, **Jas8**, **Jas9**, **Jas10**, as well as caffeine, by 50%, compared to the 6-OHDA group (Figure 9).

#### 2.4.3. Effects of Newly Synthesized Derivatives of Caffeine-8-α-Methyl-Thioglycolic Acid (**Jas1**–**Jas10**) on Isolated Rat Brain Mitochondria

**Figure 10:**
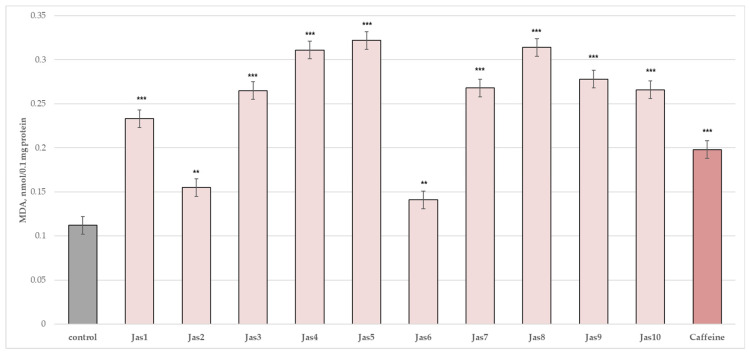
Effects of the **Jas1**–**Jas10** series and caffeine (at a concentration of 100 µM), applied individually, on MDA production in brain mitochondria. ** *p* < 0.01; *** *p* < 0.001 compared to the control (untreated brain mitochondria).

**Figure 11:**
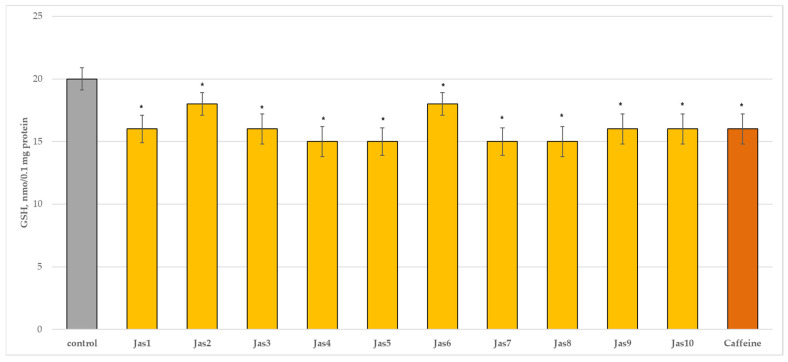
Effects of the **Jas1**–**Jas10** series and caffeine (at a concentration of 100 µM), applied individually, on glutathione (GSH) levels in brain mitochondria. * *p* < 0.05 compared to the control (untreated mitochondria).

When applied individually at a concentration of 100 µM on isolated mouse brain mitochondria, the compounds from the **Jas1**–**Jas10** series exhibited a statistically significant neurotoxic effect compared to untreated controls (Figure 10 and Figure 11).

**Figure 12:**
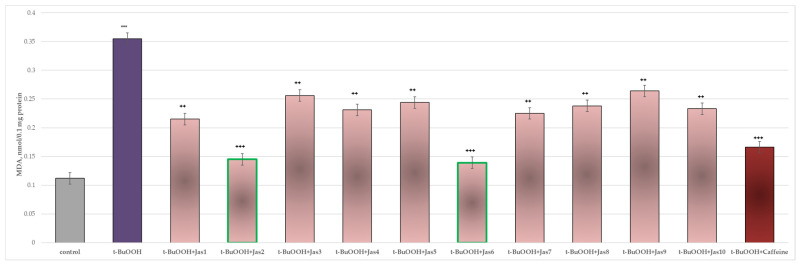
Effects of the **Jas1**–**Jas10** series and caffeine (at a concentration of 100 µM), applied individually, on MDA production in brain mitochondria under *tert*-butyl hydroperoxide-induced oxidative stress. *** *p* < 0.001 compared to the control (untreated mitochondria); ++ *p* < 0.01; +++ *p* < 0.001 compared to *t*-BuOOH.

**Figure 13:**
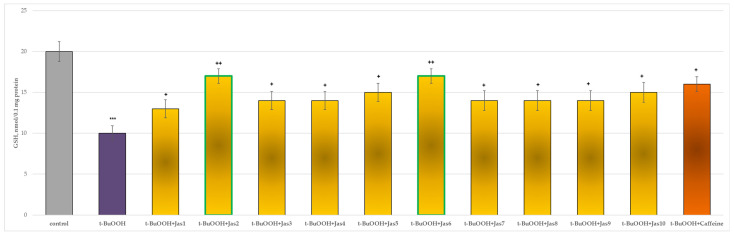
Effects of the **Jas1**–**Jas10** series and caffeine (at a concentration of 100 µM) under conditions of *tert*-butyl hydroperoxide–induced oxidative stress on the GSH level in brain mitochondria. *** *p* < 0.001 vs. control (untreated mitochondria); + *p* < 0.05; ++ *p* < 0.01 vs. *t*-BuOOH.

However, under *tert*-butyl hydroperoxide (*t*-BuOOH)-induced oxidative stress, the entire **Jas1**–**Jas10** series showed significant neuroprotective and antioxidant effects by preserving reduced glutathione (GSH) levels and reducing malondialdehyde (MDA) production relative to the neurotoxic agent (Figure 12 and Figure 13). **Jas2** and **Jas6** demonstrated the most pronounced neuroprotective and antioxidant activities in this model of neurotoxicity, while also exhibiting the lowest neurotoxicity when applied alone.

Regarding MDA production, the **Jas1**–**Jas10** compounds and caffeine applied alone caused a slight but statistically significant pro-oxidant effect. Specifically, **Jas1** increased MDA production by 108%, **Jas2** by 38%, **Jas3** by 137%, **Jas4** by 178%, **Jas5** by 188%, **Jas6** by 26%, **Jas7** by 139%, **Jas8** by 180%, **Jas9** by 148%, **Jas10** by 138%, and caffeine by 77% relative to untreated mitochondria (Figure 10).

The reductions in GSH levels were also observed with individual application of the **Jas1**–**Jas10** compounds and caffeine; **Jas1**, **Jas3**, **Jas9**, **Jas10**, and caffeine decreased GSH by 20%, **Jas2** and **Jas6** by 10%, and **Jas4**, **Jas5**, **Jas7**, and **Jas8** by 25% compared to control mitochondria (Figure 11).

In isolated mitochondria, *t*-BuOOH (75 µM) alone caused a statistically significant neurotoxic effect by reducing GSH levels by 50% and increasing MDA production by 217% relative to controls (Figure 12 and Figure 13).

Under *t*-BuOOH-induced oxidative stress, the **Jas1**–**Jas10** series demonstrated significant neuroprotective and antioxidant effects compared to the toxic agent. **Jas1** reduced MDA production by 39%, **Jas2** by 59%, **Jas3** by 28%, **Jas4** by 35%, **Jas5** by 31%, **Jas6** by 61%, **Jas7** by 37%, **Jas8** by 33%, **Jas9** by 26%, **Jas10** by 34%, and caffeine by 53% relative to *t*-BuOOH (Figure 12).

Moreover, following oxidative stress induction by *t*-BuOOH, **Jas1** preserved GSH levels by 30%, **Jas2** and **Jas6** by 70%, **Jas3**, **Jas4**, **Jas7**, **Jas8**, and **Jas9** by 40%, **Jas5** and **Jas10** by 50%, and caffeine by 60% compared to *t*-BuOOH alone (Figure 13).

#### 2.4.4. Effects of Newly Synthesized Caffeine-8-α-Methyl-Thioglycolic Acid Derivatives (**Jas1**–**Jas10**) on Isolated Mouse Brain Microsomes

**Figure 14:**
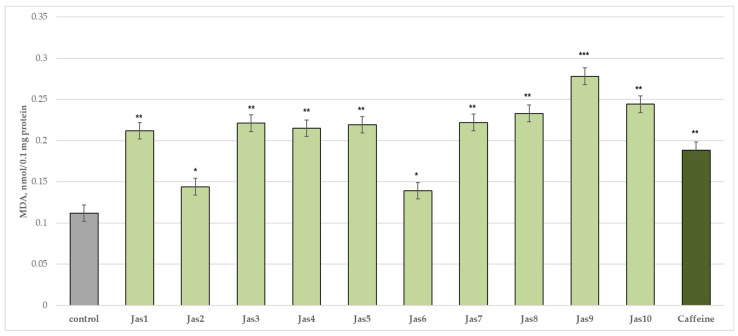
Effects of the **Jas1**–**Jas10** series and caffeine (at a concentration of 100 µM), applied individually, on MDA production in brain microsomes. * *p* < 0.05; ** *p* < 0.01; *** *p* < 0.001 vs. control (untreated brain microsomes).

When applied alone at 100 µM on isolated mouse brain microsomes, all **Jas1**–**Jas10** compounds exhibited a statistically significant pro-oxidant effect compared to untreated microsomes (Figure 14). **Jas1** increased MDA production by 89%, **Jas2** by 29%, **Jas3** by 97%, **Jas4** by 92%, **Jas5** by 96%, **Jas6** by 24%, **Jas7** by 98%, **Jas8** by 108%, **Jas9** by 148%, **Jas10** by 118%, and caffeine by 68% relative to controls.

**Figure 15:**
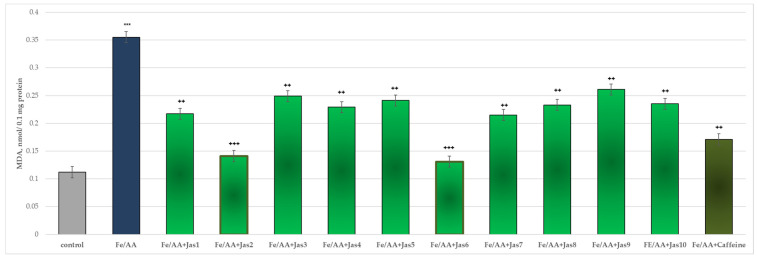
Effects of the **Jas1**–**Jas10** series and caffeine (at a concentration of 100 µM) under conditions of non-enzymatic-induced lipid peroxidation on MDA production in brain microsomes. *** *p* < 0.001 vs. control (untreated microsomes); ++ *p* < 0.01; +++ *p* < 0.001 vs. Fe/AA.

In conditions modeling non-enzymatic lipid peroxidation, however, the **Jas1**–**Jas10** series exhibited significant antioxidant activity by decreasing MDA production relative to the neurotoxic agent (Figure 15). **Jas2** and **Jas6** showed the most marked antioxidant effects in this toxicity model and displayed the weakest pro-oxidant activity when applied alone.

The iron/ascorbate (Fe/AA) combination applied alone induced a significant pro-oxidant effect, increasing MDA production by 217% compared to untreated microsomes (Figure 15).

Against this oxidative stress, **Jas1** reduced MDA production by 39%, **Jas2** by 60%, **Jas3** by 30%, **Jas4** by 35%, **Jas5** by 32%, **Jas6** by 63%, **Jas7** by 39%, **Jas8** by 34%, **Jas9** by 26%, **Jas10** by 34%, and caffeine by 52% relative to Fe/AA treatment (Figure 15).

## 3. Discussion

### 3.1. QSAR Model

The descriptors C8O and C8S indicate the presence of oxygen or sulfur atoms directly linked to the 8-position of the xanthine ring. Their positive regression coefficients suggest that a linkage between the side chain and the xanthine core mediated by these atoms is favorable for increasing inhibitory activity, likely because sulfur is more polarizable than oxygen and can strengthen hydrophobic and van der Waals interactions with the binding site. This makes S-containing linkages particularly attractive for improving affinity [23,24].

link(CH_2_)_4_ and linkO represent properties of the chain connecting the two terminal fragments. link(CH_2_)_4_ denotes the presence of a four-methylene bridge, while linkO reflects the occurrence of a secondary heteroatom (oxygen) in the chain. Their positive coefficients indicate that chain elongation and the introduction of an additional oxygen atom enhance antagonistic activity.

At the same time, the linker descriptors show that both chain length and heteroatom incorporation are important. While link(CH_2_)_4_ suggests that elongation improves activity, choosing a shorter linker with two CH_2_ groups can still maintain sufficient flexibility while avoiding excessive conformational freedom. Introducing a methyl substituent into this shorter linker can compensate for reduced length by increasing hydrophobic contact and steric complementarity within the receptor pocket [25,26].

Putting these together, compounds with sulfur at C8, a two-methylene linker, and a methyl substituent represent a balanced design: sulfur enhances core binding, the shorter CH_2_ chain preserves an optimal spatial arrangement, and the methyl group boosts hydrophobic interactions. This combination can mimic the beneficial effects of longer or oxygen-containing linkers while potentially improving selectivity and reducing unnecessary flexibility [27].

The remaining descriptors characterize structural features of the substituents opposite the xanthine core. EndC_5_H_10_ and EndCH(CH_3_)_2_ correspond to cyclopentyl and isopropyl substituents, respectively. Their negative coefficients indicate that these groups reduce MAO-B inhibitory activity. In contrast, the presence of chlorine and bromine atoms at the meta or para positions of the benzene ring has the opposite effect, as reflected by the positive coefficients for End mBr benz, End pBr benz, End mCl benz, and End pCl benz.

Four compounds, identified as outliers and marked in pink in the figure, have calculated pIC_50_ values differing by more than one logarithmic unit from the experimental ones. In all of these, the connecting bridge is absent or shorter than four methylene groups (Figure 1). Two also contain an unsubstituted phenyl residue, while one features a cyclohexyl group. These factors account for the model’s failure to accurately predict their inhibitory activity (Figure 1).

Cross-validation and Y-scrambling tests indicate that the resulting QSAR model is suitable for predicting the inhibitory activity of novel, unsynthesized compounds, thus informing the rational design of new homologs with MAO-B activity.

### 3.2. Molecular Docking

The results demonstrated that the most stable enzyme-ligand complex was formed by the ligand **JaS7**. The XP docking score of the latter compound was −12.08 kcal/mol which is comparable to the score of the selective co-crystallized molecule Safinamide (−13.45 kcal/mol). The rest of the structures containing thiosemicarbazide (**JaS2**, **JaS8** and **JaS9**) also displayed good docking scores with Glide (from −10.11 to −10.81 kcal/mol) (Table 2). The molecules containing oxygen instead of sulfur (semicarbazide moiety) demonstrated slightly lower docking scores when Glide was used. In most cases the application of both S and R chiral forms led to identical binding scores, therefore the molecules could be synthesized and tested as racemic mixtures. To improve the reliability of the acquired results GOLD 5.3 was applied for consensus docking (Table 2). The results showed that none of the xanthines included in the dataset possess a higher binding score than the standard Safinamide. An introduction of a cyclyhexyl fragment in the xanthines (ligands **JaS6** and **JaS7**) provided the highest ChemPLP scores of 77.75 and 79.37, respectively. Finally, the binding free energies of the top-scoring xanthines was calculated with MM/GBSA. Importantly, the docking scores of the top-ranked ligands **JaS6** and **JaS7** were confirmed after the MM/GBSA recalculations. Both ligands displayed the formation of stable complexes with free binding energies of −74.73 (**JaS6**) and −72.56 (**JaS7**) (Table 2).

The results showed that most of the stabilizing forces were hydrophobic in nature, which corresponds to recent findings [28,29]. Interestingly, in some of the complexes, the carbonyl group located at the sixth position in the xanthine ring was involved in H_2_O-mediated H-bonds with active amino acids (mainly Cys172 and Tyr188). Thus, the favorable active water molecule HOH1155 should be considered when conducting docking simulations of xanthine-based compounds (Figure 3). Moreover, the placement of the xanthine ring in the “aromatic cage” lead to the formation of more stable complexes due do the aforementioned water-mediated hydrogen bonds. The interaction of the top-ranked compounds with the active amino residue Cys172 is of great importance for a hypothetical MAO-B selectivity, considering the absence of the former amino acid in the active site of the crystal MAO-A enzyme [30]. Three of the top ten xanthine-based molecules were observed to interact with the active water HOH1155—**JaS1**, **JaS2**, and **JaS7**. However, none of the described compounds formed additional π−π bonds with the amino acids constructing the aromatic cage of the active gorge of MAO-B. The former bonds are important for the additional stabilization of the complexes and an enhanced MAO-B blocking activity [31]. π−π interactions were detected in the formation of the complexes with **JaS3**, **JaS4**, **JaS5**, **JaS8**, **JaS9** and **JaS10**. Further visualization of the active conformations of the hit compounds demonstrated that the active amino acid Tyr326 was not involved in any π−π stacking interactions with the xanthine derivatives. The former amino acid is essential for the stabilization, considering it separates the entrance from the substrate cavity [32]. Overall, the designed xanthine-based ligands exhibited favorable consensus docking scores and formed stable complexes with the enzyme.

Interestingly, the purine dion core structure was trapped in the aromatic cage, forming stable water-mediated H-bonds (Figure 3). Identical localization of the xanthine ring was discussed in a recent paper [33]. The position of the xanthine ring promotes the formation of hydrogen bond between the OH-group of Tyr188 and the carbonyl group at the sixth position of the core structure.

Importantly, the docking scores of the top-ranked ligands **JaS6** and **JaS7** were confirmed after the MM/GBSA recalculations (Table 2). Both ligands displayed the formation of stable complexes with free binding energies of −74.73 (**JaS6**) and −72.56 (**JaS7**).

### 3.3. Synthesis of the Target Semi- and Thiosemicarbazides

The trend observed in Table 3 can be rationalized by considering both solubility effects and the intrinsic influence of the solvent on reaction kinetics and equilibria. In aqueous media, the markedly lower yields are most likely a consequence of limited solubility of one or more reactants, leading to heterogeneous conditions and reduced effective collisions between reactive species. In addition, water can competitively stabilize intermediates or participate in side reactions (e.g., hydrolysis), further decreasing the formation of the desired product.

In contrast, the use of N,N-Dimethylformamide (DMF) provides a homogeneous reaction environment due to its strong solvating ability for both polar and moderately nonpolar substrates. As a polar aprotic solvent, DMF does not strongly hydrogen-bond to nucleophiles, thereby preserving their reactivity and often accelerating reaction rates. This can explain not only the improved yields but also the shorter reaction times observed in the first two cases.

Moreover, DMF’s relatively high boiling point allows reactions to be conducted at elevated temperatures without significant solvent loss, which can further enhance conversion. Taken together, these factors suggest that the superior performance in DMF arises from a combination of improved reactant solubility, enhanced nucleophilicity, and more favorable kinetic conditions compared to aqueous media.

### 3.4. In Vitro Assessment of Neurotoxicity and Neuroprotection

The assessment of the effects of newly synthesized derivatives of caffeine-8-α-methylthioglycolic acid (**Jas1**–**Jas10**) on isolated mouse brain synaptosomes (Figure 4 and Figure 5), mouse brain mitochondria (Figure 10 and Figure 11), and microsomes (Figure 14) under basal conditions and in the same subcellular fractions under oxidative stress conditions (Figure 6 and Figure 7 for synaptosomes; 12 and 13 for mitochondria and 15 for microsomes, respectively) is presented. In all subcellular in vitro evaluations, caffeine was used as a comparative substance. The results reveal a consistent dual effect profile, characterized by mild pro-oxidant activity in the absence of stress and significant antioxidant and neuroprotective effects under oxidative challenge of the investigated derivatives in comparison to caffeine, applied as a reference (Figure 4, Figure 5, Figure 6, Figure 7, Figure 8, Figure 9, Figure 10, Figure 11, Figure 12, Figure 13, Figure 14 and Figure 15, respectively, for synaptosomes, mitochondria and microsomes).

Under basal conditions, all compounds induced a moderate increase in malondialdehyde (MDA) levels in mitochondria and microsomes (Figure 10 and Figure 12), accompanied by a reduction in glutathione (GSH) in synaptosomes and mitochondria (Figure 5 and Figure 11). These findings indicate a mild disruption of redox homeostasis and are consistent with previous reports describing context-dependent pro-oxidant effects of caffeine and related compounds [34,35].

In contrast, under oxidative stress conditions, all derivatives demonstrated significant protective effects. In mitochondria, oxidative damage induced by *tert*-butyl hydroperoxide (t-BuOOH) was attenuated, as evidenced by reduced MDA production and preservation of GSH levels (Figure 12 and Figure 13). Similarly, in synaptosomes, 6-hydroxydopamine (6-OHDA)-induced toxicity was significantly mitigated, with improved viability and restoration of GSH levels (Figure 8 and Figure 9). In microsomes, although the compounds exhibited pro-oxidant effects when applied alone, they significantly inhibited lipid peroxidation induced by the iron/ascorbate system (Fe/AA), further confirming their antioxidant capacity in membrane systems.

Among the tested derivatives, **Jas2** and **Jas6** consistently showed the most favorable profiles, combining the lowest intrinsic toxicity with the strongest protective effects across all models. These compounds demonstrated the greatest inhibition of lipid peroxidation and the highest preservation of GSH, reaching up to 70% restoration under oxidative stress conditions. Their activity was comparable to or greater than that of caffeine.

The observed switch from pro-oxidant to antioxidant activity may be attributed to redox-modulating properties. Under basal conditions, the compounds may promote limited reactive oxygen species (ROS) generation through interactions with mitochondrial or microsomal systems. However, in the presence of strong oxidants, they likely act as radical scavengers or chain-breaking antioxidants, reducing lipid peroxidation and preserving cellular antioxidant defenses. This behavior is consistent with hormetic mechanisms described for redox-active molecules [36].

The ability to preserve GSH appears central to the protective effects observed. As a key intracellular antioxidant, GSH plays a critical role in maintaining redox balance and protecting against oxidative damage. Its depletion is strongly associated with neuronal dysfunction and neurodegenerative processes [37]. The significant preservation of GSH by **Jas2** and **Jas6** highlights their potential as modulators of endogenous antioxidant defenses.

The synaptosomal findings are particularly relevant, as synaptic terminals are highly susceptible to oxidative stress. Protection against 6-hydroxydopamine-induced damage suggests potential efficacy in models of neurodegeneration, including Parkinson’s disease. In parallel, the inhibition of lipid peroxidation in microsomes supports the membrane-stabilizing properties of the compounds.

Overall, although the **Jas1**–**Jas10** derivatives exhibit mild pro-oxidant effects under basal conditions, their consistent antioxidant and neuroprotective effects under oxidative stress across multiple subcellular systems highlight their potential as neuroprotective agents. Further studies are needed to elucidate their mechanisms of action and to confirm their efficacy in vivo.

These findings are in line with existing literature demonstrating the neuroprotective potential of methylxanthine derivatives, including caffeine and its metabolites, which act via multiple mechanisms such as reducing oxidative stress and modulating intracellular signaling pathways relevant to neuronal survival. In addition, the identified superior *h*MAOB selectivity indices of **Jas2** and **Jas6** highlight their promise as potent MAO-B inhibitors, an established therapeutic target in neurodegenerative diseases like Parkinson’s disease. Their dual neuroprotective and antioxidant actions suggest beneficial modulation of mitochondrial function and reactive oxygen species, key contributors to neuronal damage.

Given the broad pharmacological spectrum of methylxanthines and their documented protective effects against neurodegeneration, cardiovascular damage, and metabolic disturbances, the mild neurotoxicity observed warrants cautious optimization in future development. Nonetheless, **Jas2** and **Jas6** emerge as lead candidates for in-depth in vitro and in vivo exploration, aiming to leverage their MAO-B inhibitory potential alongside antioxidant properties for the development of novel neuroprotective agents.

## 4. Materials and Methods

### 4.1. QSAR Model

A total of 94 compounds were selected from the literature [14,15,16,17,18], each featuring a 1,3,7-methyl- or ethyl-substituted xanthine moiety connected at position 8 via an ether or thioether bridge to cyclic (aromatic or non-aromatic) or aliphatic substituents. Their inhibitory activities against MAO-B were determined as IC_50_ (the concentration required to inhibit 50% of enzyme activity), with measurements performed in the same laboratory using the same experimental protocol, in accordance with good QSAR practice. For the purposes of the QSAR analysis, the effect of each compound was expressed as pIC_50_ (pIC_50_ = −log IC_50_), whereby higher pIC_50_ values correspond to more potent inhibitors. The pIC_50_ values span four logarithmic units, indicating the presence of both weak and highly active compounds in the series, thus enabling the development of a statistically significant QSAR model.

The molecular structures were constructed and visualized using the ACDLabs software v.9.08 as an initial step in their preparation for analysis. Structural optimization was subsequently performed using molecular mechanics (MM+) and semi-empirical quantum chemical (AM1) methods via HyperChem v.7.5.

### 4.2. Selection and Preparation of Proteins

The X-ray crystal of MAO-B with PDB code **2V5Z**, resolved with a co-crystallized selective inhibitor, Safindamide, was downloaded from the Protein Data Bank (PDB). The Protein Preparation Wizard in Maestro [38] was employed for the protein refinements. Hydrogen bonds and het states at pH 7.0 were generated. The active waters of MAO-B were preserved for the docking studies. Moreover, the conformational energy of the crystal structure was minimized by the OPLS4 force field.

### 4.3. Preparation of Ligands

The structures were prepared for the virtual screening with the LigPrep module (Schrödinger Release 2021-3: LigPrep, Schrödinger, LLC, New York, NY, USA, 2021) [39]. Utilizing LigPrep, hydrogen bonds, tautomers and ionization states at pH 7.0 were generated. Furthermore, the energies of the ligands were minimized by applying the OPLS4 force field.

### 4.4. Docking Protocols

The docking programs Glide (Maestro) and GOLD 5.3 were applied for the molecular docking calculations. Glide uses an empirically based GlideScore scoring algorithm, which is presented in three forms: High-throughput screening (HTS), Standard-Precision (SP) and Extra-Precision (XP) modes. Initially, HTVS was applied for the virtual screening. Thereafter, the most precise docking scoring mode of Maestro was used—XP. GOLD 5.3 was applied as a second docking software to increase the reliability of the acquired Glide scores. The default scoring option ChemPLP was introduced in the theoretical calculations. Finally, the binding free energies of the top-scoring complexes were calculated with the MM/GBSA Prime module.

### 4.5. Synthesis

All commercially available chemicals and solvents used were of analytical grade and purchased from standard commercial suppliers such as Merck (N,N-dimethylformamide (DMF), mercaptans, hydrochloric acid (HCl)), Fluka (bromine, ethanol, sodium hydroxide (NaOH), methanol), and Sigma-Aldrich (1,3,7-trimethylxanthine, hydrazine hydrate, hdrazinecarboxamides and hydrazinthyocarboxamides). Reagents were used without further purification unless otherwise stated.

#### 4.5.1. Synthesis of 8-Bromocaffeine

The initial xanthine derivatives were synthesized by bromination of 1,3,7-trimethylxanthine under controlled conditions. Typically, 1,3,7-trimethylxanthine was reacted with bromine in water or hydrobromic acid medium at room temperature to yield 8-bromoxanthine derivatives. Reaction progress was monitored by TLC.

#### 4.5.2. Synthesis of Caffeine-8-α-Methylthioglycolic Acid

Two synthetic approaches were tested. The first used 8-bromocaffeine and mercaptans in DMF at 140 °C for 12 h, yielding 9%, with side hydrolysis suspected. The second, improved method involved reacting the sodium mercaptan salt with 8-bromocaffeine in refluxing aqueous ethanol with NaOH, which afforded pure product in 99–100% yield after 1 h.

#### 4.5.3. Synthesis of Methyl Ester of Caffeine 8-α-Methylthioglycolic Acid

The methyl ester was prepared by reacting the in situ-generated acid chloride of caffeine 8-α-methylthioglycolic acid with methanol. This method afforded high yields (82–96%) within 30 min to 1 h under reflux conditions (60 °C). The absence of side products and gaseous byproducts (SO_2_, HCl) facilitated product isolation, making this approach efficient for obtaining the ester for further synthesis.

#### 4.5.4. Synthesis of Caffeine 8-α-Methylthioglycolic Acid Hydrazide

Direct reaction of caffeine 8-α-methylthioglycolic acid with hydrazine hydrate was unsuccessful due to a lack of reaction below 70 °C and product decomposition above 100 °C. Instead, the hydrazide was obtained by hydrazinolysis of the methyl ester of caffeine 8-α-methylthioglycolic acid. This reaction proceeded smoothly upon refluxing in an alcoholic medium for 2 h, yielding 73%.

#### 4.5.5. Synthesis of Xanthine-Based Semi- and Thiosemicarbazides

The synthesis proceeded via the reaction of eight substituted xanthine derivatives with appropriate hydrazinecarboxamide or hydrazinecarbothioamide derivatives. The reactions were carried out using a molar ratio of reactants of 1:1.25.

Reactions were conducted in suitable solvents, primarily dimethylformamide (DMF) or water, at temperatures ranging from ambient to 140 °C, depending on the specific step. Reaction progress was monitored by thin-layer chromatography (TLC) using appropriate solvent systems until complete consumption of the starting material was observed.

After completion, the reaction mixtures were cooled to room temperature, and the products were isolated by standard work-up procedures, including solvent removal under reduced pressure and purification by crystallization or chromatographic techniques.

The corresponding structural features, measured through appropriate spectral methods, including IR, ^1^H and ^13^C NMR and LC-MS techniques, together with the IUPAC names of the new products are presented below:

*2-(2-((1,3,7-trimethyl-2,6-dioxo-2,3,6,7-tetrahydro-1H-purin-8-yl)thio)propanoyl)hydrazine-1-carbothioamide* (**Jas2**): FTIR (ATR, cm^−1^): 3202 (νNH); 1708 (νCO–xanthine); 1679 (νCO–xanthine);1651 with shoulder at 1663 (νCO–amide I);1602, 1537 (νC=N, νC=C–xanthine, *δ*NH–amide II); ^1^H NMR (*δ*H, 400 MHz, DMSOd_6_): δ 1.50 [d, 3H, CH_3_], 3.35 [s, 3H, N-CH_3_], 3.41 [s, 3H, N-CH_3_], 3.78 [s, 3H, N-CH_3_], 3.93 [q, 1H, CH–CH_3_], 7.68 [s, 1H, CO–NH], 9.53 [s, 2H, NH_2_], 10.40 [br s, 1H, NH]; ^13^C NMR (100 MHz, DMSOd_6_): δ 18.7, 29.0, 30.7, 35.3, 43.6, 107.1, 148.7, 151.3, 151.7, 154.8, 174.8, 182.5, LC-MS (70 eV) *m*/*z* (%) 356 (M+), recrystallized from water/ethanol mixture.

*N-phenyl-2-(2-((1,3,7-trimethyl-2,6-dioxo-2,3,6,7-tetrahydro-1H-purin-8-yl)thio)propanoyl) hydrazine-1-carboxamide* (**Jas3**): FTIR (ATR, cm^−1^): 3205 (νNH); 1708 (νCO–xanthine); 1648 with shoulders at 1668 and 1651 (νCO–xanthine, νCO–amide I);1604, 1532 (νC=N, νC=C–xanthine, δNH–amide II); ^1^H NMR (δH, 400 MHz, DMSOd_6_): δ 1.50 [d, 3H, CH_3_], 3.35 [s, 3H, N–CH_3_], 3.41 [s, 3H, N–CH_3_], 3.78 [s, 3H, N–CH_3_], 3.93 [q, 1H, CH–CH_3_], 7.07 [t, 1H, Ar–H(4′)], 7.37 [t, 1H, Ar–H(3′)], 7.37 [t, 1H, Ar-H(5′)], 7.50 [d, 1H, Ar–H(2′)], 7.50 [d, 1H, Ar-H(6′)], 9.26 [s, 1H, CO-NH–Ar], 10.08 [s, 1H, CO-NH], 11.03 [s, 1H, NH]; ^13^C NMR (100 MHz, DMSOd_6_): δ 18.7, 29.0, 30.7, 35.3, 42.6, 107.1, 121.6 (2C), 128.0, 128.9 (2C), 139.4, 148.7, 151.3, 151.7, 153.8, 154.8, 174.8, LC-MS (70 eV) *m*/*z* (%) 372 (M+), recrystallized from water/ethanol mixture.

*N-(3-chlorophenyl)-2-(2-((1,3,7-trimethyl-2,6-dioxo-2,3,6,7-tetrahydro-1H-purin-8-yl)thio) propanoyl)hydrazine-1-carboxamide* (**Jas4**): FTIR (ATR, cm^−1^): 3189 (νNH); 1707 (νCO–xanthine); 1646 with shoulder at 1666 and 1656 (νCO–xanthine, νCO–amide I); 1604, 1533(νC=N, νC=C–xanthine, δNH–amide II); ^1^H NMR (δH, 400 MHz, DMSOd_6_): δ 1.50 [d, 3H, CH_3_], 3.35 [s, 3H, N–CH_3_], 3.41 [s, 3H, N–CH_3_], 3.78 [s, 3H, N–CH_3_], 3.93 [q, 1H, CH–CH_3_], 7.16 [d, 1H, Ar–H(4′)], 7.39 [t, 1H, Ar–H(5′)], 7.46 [t, 1H, Ar–H(6′)], 7.90 [d, 1H, Ar–H(2′)], 9.26 [s, 1H, CO-NH–Ar], 10.08 [s, 1H, CO-NH], 11.03 [s, 1H, NH]; ^13^C NMR (100 MHz, DMSOd_6_): δ 18.7, 29.0, 30.7, 35.3, 42.6, 107.1, 119.7, 122.0, 127.9, 130.3, 134.5, 138.4, 148.7, 151.3, 151.7, 153.8, 154.8, 174.8, LC-MS (70 eV) *m*/*z* (%) 432 (M+), recrystallized from ethanol.

*N-(4-chlorophenyl)-2-(2-((1,3,7-trimethyl-2,6-dioxo-2,3,6,7-tetrahydro-1H-purin-8-yl)thio) propanoyl)hydrazine-1-carboxamide* (**Jas5**): FTIR (ATR, cm^−1^): 3193 (νNH); 1705 (νCO–xanthine); 1670, 1648 (νCO–xanthine, νCO–amide I); 1602, 1550 with shoulder at 1540 (νC=N, νC=C–xanthine, δNH–amide II); ^1^H NMR (δH, 400 MHz, DMSOd_6_): δ 1.50 [d, 3H, CH_3_], 3.35 [s, 3H, N–CH_3_], 3.41 [s, 3H, N–CH_3_], 3.78 [s, 3H, N–CH_3_], 3.93 [q, 1H, CH–CH_3_], 7.40 [d, 1H, Ar-H(3′)], 7.40 [d, 1H, Ar-H(5′)], 7.72 [d, 1H, Ar-H(2′)], 7.72 [d, 1H, Ar-H(6′)], 9.26 [s, 1H, CO-NH–Ar], 10.08 [s, 1H, CO-NH], 11.03 [s, 1H, NH]; ^13^C NMR (100 MHz, DMSOd_6_): δ 18.7, 29.0, 30.7, 35.3, 42.6, 107.1, 120.8 (2C), 129.0 (2C), 133.3, 137.5, 148.7, 151.3, 151.7, 153.8, 154.8, 174.8 LC-MS (70 eV) *m*/*z* (%) 467 (M+), recrystallized from water/ethanol mixture.

*N-cyclohexyl-2-(2-((1,3,7-trimethyl-2,6-dioxo-2,3,6,7-tetrahydro-1H-purin-8-yl)thio)propanoyl) hydrazine-1-carboxamide* (**Jas6**): FTIR (ATR, cm^−1^): 3199 (νNH); 1699 (νCO–xanthine); 1660 with shoulder at 1668 and 1664 (νCO–xanthine, νCO–amide I); 1607, 1533(νC=N, νC=C–xanthine, δNH–amide II); ^1^H NMR (δH, 400 MHz, DMSOd_6_): δ 1.11–1.21 [m, 2H, cyclohexyl-CH_2_(3′)], 1.11–1.21 [m, 2H, cyclohexyl-CH_2_(5′)], 1.44–1.46 [m, 2H, cyclohexyl-CH_2_(4′)], 1.49–1.74 [m, 2H, cyclohexyl-CH_2_(2′)], 1.49–1.74 [m, 2H, cyclohexyl-CH_2_(6′)], 1.50 [d, 3H, CH_3_], 3.35 [s, 3H, N–CH_3_], 3.41 [s, 3H, N–CH_3_], 3.54 [m, 1H, cyclohexyl-CH], 3.78 [s, 3H, N–CH_3_], 3.93 [q, 1H, CH–CH_3_], 6.46 [s, 1H, CO-NH–cyclohexyl], 10.08 [s, 1H, CO-NH], 11.03 [s, 1H, NH]; ^13^C NMR (100 MHz, DMSOd_6_): δ 18.7, 24.8 (2C), 25.7, 29.0, 30.7, 32.7 (2C), 35.3, 42.6, 53.5, 107.1, 148.7, 151.3, 151.7, 154.8, 158.4, 174.8, LC-MS (70 eV) *m*/*z* (%) 438 (M+), recrystallized from water/ethanol mixture.

*N-cyclohexyl-2-(2-((1,3,7-trimethyl-2,6-dioxo-2,3,6,7-tetrahydro-1H-purin-8-yl)thio)propanoyl) hydrazine-1-carbothioamide* (**Jas7**): FTIR (ATR, cm^−1^): 3203 (νNH); 1697 (νCO–xanthine); 1655 with shoulder at 1635 and 1599 (νCO–xanthine, νCO–amide I); 1538 with shoulder at 1546 (νC=N, νC=C–xanthine, δNH–amide II); ^1^H NMR (δH, 400 MHz, DMSOd_6_): δ 1.11–1.21 [m, 2H, cyclohexyl-CH_2_(3′)], 1.11–1.21 [m, 2H, cyclohexyl-CH_2_(5′)], 1.44–1.46 [m, 2H, cyclohexyl-CH_2_(4′)], 1.47–1.72 [m, 2H, cyclohexyl-CH_2_(2′)], 1.47–1.72 [m, 2H, cyclohexyl-CH_2_(6′)], 1.50 [d, 3H, CH_3_], 2.57 [m, 1H, cyclohexyl-CH], 3.35 [s, 3H, N–CH_3_], 3.41 [s, 3H, N–CH_3_], 3.78 [s, 3H, N–CH_3_], 3.93 [q, 1H, CH–CH_3_], 7.31 [s, 1H, CS-NH-cyclohexyl], 7.68 [s, 1H, CO-NH], 10.40 [br s, 1H, NH]; ^13^C NMR (100 MHz, DMSOd_6_): δ 18.7, 25.4 (2C), 25.7, 29.0, 30.7, 32.5 (2C), 35.3, 43.6, 58.0, 107.1, 148.7, 151.3, 151.7, 154.8, 174.8, 184.2, LC-MS (70 eV) *m*/*z* (%) 454 (M^+^)/468, normal phase silica gel stationary phase chromatography with ethyl acetate/methanol = 15/5 *v*/*v* as a mobile phase.

*N-(4-bromocyclohexyl)-2-(2-((1,3,7-trimethyl-2,6-dioxo-2,3,6,7-tetrahydro-1H-purin-8-yl)thio) propanoyl)hydrazine-1-carbothioamide* (**Jas8**): FTIR (ATR, cm^−1^): 3200 (νNH); 1700 (νCO–xanthine); 1656 with shoulder at 1670 (νCO–xanthine, νCO–amide I); 1603, 1537 with shoulder at 1506 (νC=N, νC=C–xanthine, δNH–amide II); ^1^H NMR (δH, 400 MHz, DMSOd_6_): δ 1.47–1.72 [m, 2H, cyclohexyl-CH_2_(2′)], 1.47–1.72 [m, 2H, cyclohexyl-CH_2_(6′)], 1.50 [d, 3H, CH_3_], 1.77–2.02 [m, 2H, cyclohexyl-CH_2_(3′)], 1.77–2.02 [m, 2H, cyclohexyl-CH_2_(5′)], 2.57 [m, 1H, cyclohexyl-CH], 3.35 [s, 3H, N–CH_3_], 3.41 [s, 3H, N–CH_3_], 3.74 [m, 1H, cyclohexyl-CH-Br], 3.78 [s, 3H, N–CH_3_], 3.93 [q, 1H, CH–CH_3_], 7.31 [s, 1H, CS-NH-cyclohexyl], 7.68 [s, 1H, CO-NH], 10.40 [br s, 1H, NH]; ^13^C NMR (100 MHz, DMSOd_6_): δ 18.7, 29.0, 30.2 (2C), 30.7, 35.2, 37.5 (2C), 43.6, 53.0, 57.3, 107.1, 148.7, 151.3, 151.7, 154.8, 174.8, 184.2, LC-MS (70 eV) *m*/*z* (%) 533 (M+), recrystallized from water/ethanol mixture.

*N-phenyl-2-(2-((1,3,7-trimethyl-2,6-dioxo-2,3,6,7-tetrahydro-1H-purin-8-yl)thio)propanoyl) hydrazine-1-carbothioamide* (**Jas9**): FTIR (ATR, cm^−1^): 3207 (νNH); 1697 with shoulder at 1704 (νCO–xanthine); 1655 with shoulder at 1637 and 1619 (νCO–xanthine, νCO–amide I); 1597, 1538 with shoulder at 1550 (νC=N, νC=C–xanthine, δNH–amide II); ^1^H NMR (δH, 400 MHz, DMSOd_6_): δ 1.50 [d, 3H, CH_3_], 3.35 [s, 3H, N–CH_3_], 3.41 [s, 3H, N–CH_3_], 3.78 [s, 3H, N–CH_3_], 3.93 [q, 1H, CH–CH_3_], 7.09 [t, 1H, Ar–H(4′)], 7.43 [t, 1H, Ar–H(3′)], 7.37 [t, 1H, Ar-H(5′)], 7.70 [d, 1H, Ar–H(2′)], 7.50 [d, 1H, Ar-H(6′)], 7.68 [s, 1H, CO-NH], 10.40 [br s, 1H, NH], 11.32 [s, 1H, CS-NH–Ar]; ^13^C NMR (100 MHz, DMSOd_6_): δ 18.7, 29.0, 30.7, 35.3, 43.6, 107.1, 126.5 (2C), 128.4, 129.0 (2C), 138.5, 148.7, 151.3, 151.7, 154.8, 174.8, 181.1, LC-MS (70 eV) *m*/*z* (%) 449 (M+), normal phase silica gel stationary phase chromatography with ethyl acetate/methanol = 15/5 *v*/*v* as a mobile phase.

*N-(tert-butyl)-2-(2-((1,3,7-trimethyl-2,6-dioxo-2,3,6,7-tetrahydro-1H-purin-8-yl)thio)propanoyl) hydrazine-1-carbothioamide* (**Jas10**): FTIR (ATR, cm^−1^): 3172 (νNH); 1704 (νCO–xanthine); 1686 with shoulder at 1672 and 1661 (νCO–xanthine, νCO–amide I); 1605, 1557, 1538 (νC=N, νC=C–xanthine, δNH–amide II); ^1^H NMR (δH, 400 MHz, DMSOd_6_): δ 1.37 [s, 3H, *tert*-butyl-CH_3_], 1.37 [s, 3H, *tert*-butyl-CH_3_], 1.37 [s, 3H, *tert*-butyl-CH_3_], 1.50 [d, 3H, CH_3_], 3.35 [s, 3H, N–CH_3_], 3.41 [s, 3H, N–CH_3_], 3.78 [s, 3H, N–CH_3_], 3.93 [q, 1H, CH–CH_3_], 7.31 [s, 1H, CS-NH-*tert*-butyl], 7.68 [s, 1H, CO-NH], 10.40 [br s, 1H, NH]; ^13^C NMR (100 MHz, DMSOd_6_): δ 18.7, 29.0, 29.4 (3C), 30.7, 35.3, 43.6, 61.0, 107.1, 148.7, 151.3, 151.7, 154.8, 174.8, 184.2, LC-MS (70 eV) *m*/*z* (%) 428 (M+), normal phase silica gel stationary phase chromatography with ethyl acetate/methanol = 15/5 *v*/*v* as a mobile phase.

### 4.6. Biological Evaluations

Monoamine oxidase (MAO) activity for both recombinant human MAO-A (*h*MAOA) and MAO-B (*h*MAOB) was assessed using a fluorometric assay based on the Amplex^®^ UltraRed reagent (Invitrogen™, Thermo Fisher Scientific, Waltham, MA, USA). This method, originally reported by Bautista-Aguilera et al. [21] and later adapted with minor changes by Kasabova-Angelova et al. [22], detects hydrogen peroxide or peroxidase activity present in enzymes or biological samples. In this assay, Amplex^®^ Red reacts in a 1:1 molar ratio with hydrogen peroxide in the presence of peroxidase, producing the red-fluorescent compound resorufin. Due to resorufin’s strong extinction coefficient (58,000 ± 5000 cm^−1^·M^−1^), measurements could be accurately taken using either fluorometric or spectrophotometric methods. This setup permits detection of extremely low hydrogen peroxide concentrations, down to the picomolar range (approximately 10 pM in a 100 µL volume). The control conditions included the use of purified *h*MAOA or *h*MAOB in reaction buffer, enzyme solutions with hydrogen peroxide, and buffer alone. All the test compounds were initially prepared at a 1 µM final concentration. These compounds were combined with either *h*MAOA or *h*MAOB and loaded into a 96-well plate (with eight rep-licates per test compound), followed by incubation for 30 min at 37 °C in the dark. Subsequently, a 50 µL detection mix was added to each well. This mix contained Amplex^®^ Red, horseradish peroxidase (HRP), and tyramine substrate, all prepared in a reaction buffer. As this enzymatic reaction proceeded continuously, the fluorescence signal was recorded every 30 min (at 0, 30, 60, 90, 120, and 150 min) to track enzymatic kinetics. Readings were taken in dark conditions while the plates were agitated and maintained at 37 °C. Fluorescence was measured using a Synergy 2 Microplate Reader at wavelengths of 570 nm and 690 nm.

### 4.7. Isolation and Incubation of Synaptosomes

Synaptosomes were isolated via subcellular fractionation using a Percoll gradient [40]. Brain homogenates were prepared in Buffer A (5 mM HEPES, 0.32 M sucrose, pH 7.4) and centrifuged twice at 1000 rpm for 5 min at 4 °C. Supernatants were combined and centrifuged thrice at 10,000 rpm for 20 min at 4 °C to purify synaptosomal fractions.

Synaptosomes were separated on a Percoll gradient prepared with 90%, 16%, and 10% Percoll solutions. After centrifugation at 15,000 rpm for 20 min at 4 °C, synaptosomes formed a distinct middle layer and were collected, washed with Buffer B (containing NaCl, MgCl_2_, KCl, CaCl_2_, NaH_2_PO_4_, and HEPES, pH 7.4) supplemented with glucose, and centrifuged for buffer exchange. Incubations with test compounds lasted 1 h.

### 4.8. Dopaminergic Neurotoxicity Model

Synaptosomes were exposed to 150 μM 6-hydroxydopamine (6-OHDA) for 1 h to simulate neurodegenerative oxidative stress [41].

### 4.9. MTT Assay for Synaptosomal Viability

MTT (0.5 mg/mL) reduction was measured spectrophotometrically at 580 nm after incubation with synaptosomes and treatments, reflecting mitochondrial dehydrogenase activity [42,43]. The absorbance ratio of treated samples to untreated controls was expressed as percent viability.

### 4.10. Glutathione (GSH) Measurement

GSH levels were quantified by determining non-protein thiol groups after protein precipitation with trichloroacetic acid and reaction with DTNB at 412 nm [44]. Samples were centrifuged and neutralized before measurement.

### 4.11. Statistical Analysis

Synaptosomal data were analyzed with MEDCALC using the Mann–Whitney test at significance levels of *p* < 0.05, 0.01, and 0.001. Neuro2A results were processed using GraphPad Prism 5.0.

## 5. Conclusions

In conclusion, ten novel semi- and thiosemicarbazide derivatives of caffeine were successfully synthesized and, based on prior QSAR modeling and molecular docking, identified as potential selective monoamine oxidase B (MAO-B) inhibitors. The biological evaluation confirmed these predictions, demonstrating that the **JaS1**–**JaS10** series exerts measurable antioxidant and neuroprotective effects across synaptosomal, mitochondrial, and microsomal models of oxidative stress.

Quantitatively, caffeine showed consistently lower or comparable MAO-A inhibition (≈25% vs. up to 30% for **JaS** compounds) and weaker MAO-B inhibition (≈30% vs. up to 50% for the most active derivatives). Under oxidative stress conditions, caffeine also provided more limited neuroprotection, preserving GSH by ~50–60% depending on the model, whereas the most active **JaS** derivatives preserved GSH by up to ~70% and reduced MDA formation more efficiently (up to ~63% reduction vs. ~52–53% for caffeine). In addition, caffeine exhibited a more pronounced intrinsic pro-oxidant profile, increasing MDA levels by ~68–77% and reducing GSH by ~20–50%, compared with several **JaS** derivatives that showed either weaker pro-oxidant effects or a more favorable balance between basal and stress conditions.

Overall, **JaS2** and **JaS6** emerged as the most promising candidates, combining stronger MAO inhibition, improved redox protection under stress, and comparatively lower intrinsic oxidative liability, supporting their further development as multifunctional agents targeting MAO-B-related neurodegeneration.

## Figures and Tables

**Table 1:** Overview of selected structural descriptors for the QSAR model build-up.

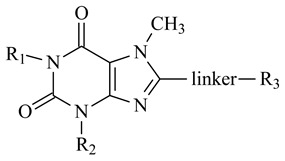					
R_1_, R_2_	Descriptor	Linker	Descriptor	R_3_	Descriptor
CH_3_-	XantCH_3_	-CH_2_-	linkCH_2_	m-F-phenyl	EndmF benz
C_2_H_5_-	XantC_2_H_5_	-(CH_2_)_2_-	Link(CH_2)2_	m-Cl-phenyl	EndmCL benz
		-(CH_2_)_3_-	Link(CH_2)3_	m-Br-phenyl	EndmBr benz
		-(CH_2_)_4_-	link(CH_2_)_4_	m-CH_3_-phenyl	EndmCH3 benz
		-O-	linkO	m-OCH_3_-phenyl	EndmOCH3 benz
		-S-	linkS	m-CF_3_-phenyl	EndmCF3 benz
		-CH_2_-O-	linkCH_2_O	p-F-phenyl	EndpF benz
		-(CH_2_)_2_-O-	link(CH_2_)_2_O	p-Cl-phenyl	EndpCL benz
		-(CH2)_4_-O-	link(CH_2_)_4_O	p-Br-phenyl	EndpBr benz
		-(CH_2_)_2_-O-CH_2_-	link(CH_2_)_2_OCH_2_	p-CH_3_-phenyl	EndpCH_3_ benz
		-CH_2_-S-	linkCH_2_S	p-OCH_3_-phenyl	EndpOCH_3_ benz
		-(CH_2_)_2_-S-	Link(CH_2_)_2_S	p-CF_3_-phenyl	EndpCF_3_ benz
				p-I-phenyl	EndpI benz
				Phenyl	EndC_6_H_6_
Heteroatom	Descriptor			m, p-diCH_3_-phenyl	Endm,pCH3 benz
-O-	C8O			Cyclohex-1-en-3-yl	EndC_6_H_10_
-S-	C8S			Cyclohexyl	EndC_6_H_12_
-NH-	C8NH			Cyclopenthyl	EndC_5_H_10_
-NHCH_3_-	C8NCH3			Isopropyl	EndCH(CH_3_)_2_
-SO-	C8S=O			2-naphtyl	EndNaphthyl
				Pyridine-2-yl	End2Pyridine
				p-etoxyphenyl	EndmC2H5O

**Table 2:** Docking scores of the designed xanthine-based compounds.

	Top Scored Compounds										
	JaS1	JaS2	JaS3	JaS4	JaS5	JaS6	JaS7	JaS8	JaS9	JaS 10	Safinamide
XP Glide (Kcal/mol)	−10.24	−10.81	−10.93	−9.82	−10.11	−11.15	−12.08	−10.48	−10.11	−10.46	−13.45
GOLD 5.3 ChemPLP	76.45	69.54	66.16	74.46	64.73	77.75	79.37	66.81	70.72	71.84	88.62
MM/GBSA	−55.14	−61.94	−51.79	−53.82	−52.74	−74.73	−72.56	−69.31	−68.61	−70.27	−83.64

**Table 3:** Reaction times, solvents and yields for the new semi- and thiosemicarbazides.

Code	Solvent	Reaction Time	Yield %
**JαS1**	Water	3 h	37
**JαS2**	Water	4 h	30
**JαS3**	DMF	1 h	99
**JαS4**	DMF	2 h	99
**JαS5**	DMF	2 h	98
**JαS6**	DMF	20 min	84
**JαS7**	DMF	6 h	71
**JαS8**	DMF	2 h	93
**JαS9**	DMF	4 h	81
**JαS10**	DMF	4 h	86

**Table 4:** IC_50_ (EC_50_) of **Jas1**–**Jas10**, Selegiline, and Chlorgyline towards *h*MAOA/B.

Compound	IC_50_ (EC_50_), (µM ± SD) *h*MAOB	IC_50_ (EC_50_), (µM ± SD) *h*MAOA	SI
**Jas1**	0.445 ± 0.10	0.613 ± 0.10	1.38
**Jas2**	0.421 ± 0.10	0.709 ± 0.09	1.68
**Jas3**	0.501 ± 0.09	0.662 ± 0.10	1.32
**Jas4**	0.516 ± 0.09	0.602 ± 0.09	1.17
**Jas5**	0.455 ± 0.09	0.711 ± 0.10	1.56
**Jas6**	0.338 ± 0.10	0.552 ± 0.10	1.63
**Jas7**	0.354 ± 0.10	0.504 ± 0.10	1.42
**Jas8**	0.366 ± 0.10	0.543 ± 0.10	1.48
**Jas9**	0.335 ± 0.09	0.499 ± 0.10	1.49
**Jas10**	0.331 ± 0.09	0.491 ± 0.10	1.48
**Selegiline**	0.220 ± 0.10	-	
**Chlorgyline**	-	0.187 ± 0.09	

SI: *h*MAOB selectivity index = IC_50_(*h*MAOA)/IC_50_(*h*MAOB).

## Data Availability

The original contributions presented in the study are included in the article and Supplementary Materials; further inquiries may be directed to the corresponding author.

## Supplementary Materials

The following supporting information can be downloaded at: https://www.mdpi.com/article/10.3390/ph19060892/s1, Figure S1. 1H NMR spectrum of JaS2; Figure S2. 1H NMR spectrum of JaS3; Figure S3. 1H NMR spectrum of JaS4; Figure S4. 1H NMR spectrum of JaS5; Figure S5. 1H NMR spectrum of JaS6; Figure S6. 1H NMR spectrum of JaS7; Figure S7. 1H NMR spectrum of JaS8; Figure S8. 1H NMR spectrum of JaS9; Figure S9. 1H NMR spectrum of JaS10; Figure S10. 13C NMR spectrum of JaS2; Figure S11. 13C NMR spectrum of JaS3; Figure S12. 13C NMR spectrum of JaS4; Figure S13. 13C NMR spectrum of JaS5; Figure S14. 13C NMR spectrum of JaS6; Figure S15. 13C NMR spectrum of JaS7; Figure S16. 13C NMR spectrum of JaS8; Figure S17. 13C NMR spectrum of JaS9; Figure S18. 13C NMR spectrum of JaS10; Figure S19. LC-MS spectrum of JaS2; Figure S20. LC-MS spectrum of JaS3; Figure S21. LC-MS spectrum of JaS4; Figure S22. LC-MS spectrum of JaS5; Figure S23. LC-MS spectrum of JaS6; Figure S24. LC-MS spectrum of JaS7; Figure S25. LC-MS spectrum of JaS8; Figure S26. LC-MS spectrum of JaS9; Figure S27. LC-MS spectrum of JaS10; Table S1: Active monoamine oxidase B amino residues participating in the interactions with the top-ranked xanthine derivatives and Safinamide.
