# Supplementary material for: Application of In Silico QSAR and Molecular Docking Studies to a Series of Xanthine-Based Analogues and Design, Synthesis and Pharmacological Evaluation of Identified New Potential Selective MAO-B Inhibitors
Source: Pharmaceuticals (Basel). 2026 Jun 4;19(6):892. doi: 10.3390/ph19060892 (PMC13305223; doi:10.3390/ph19060892)
Supplement: Supplementary file 1 [file pharmaceuticals-19-00892-s001.zip › pharmaceuticals-4273224-supplementary.pdf]

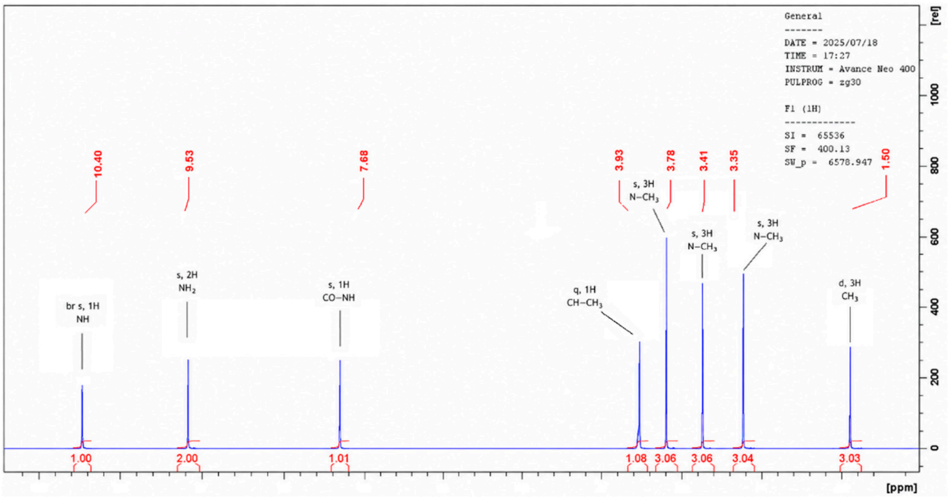

Supplementary Figure S1. ^{1}H NMR spectrum of JaS2.

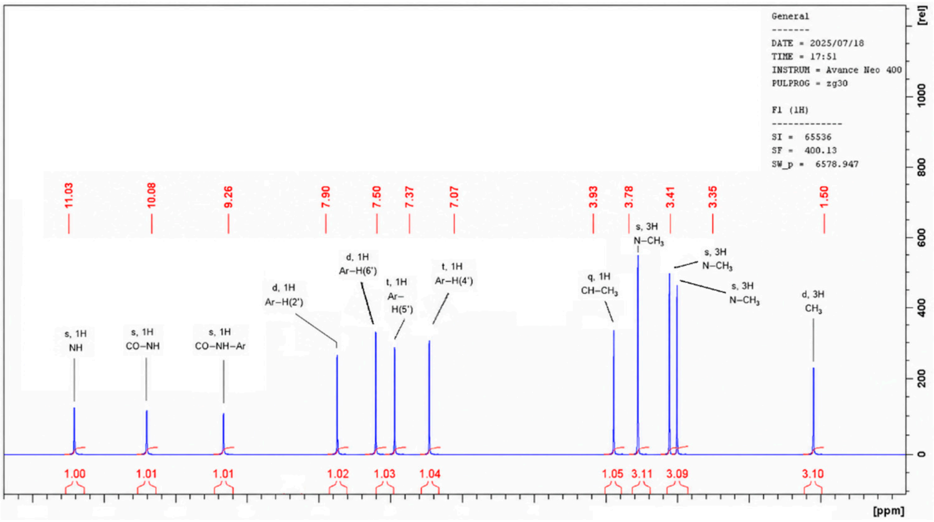

Supplementary Figure S2. ^{1}H NMR spectrum of JaS3.

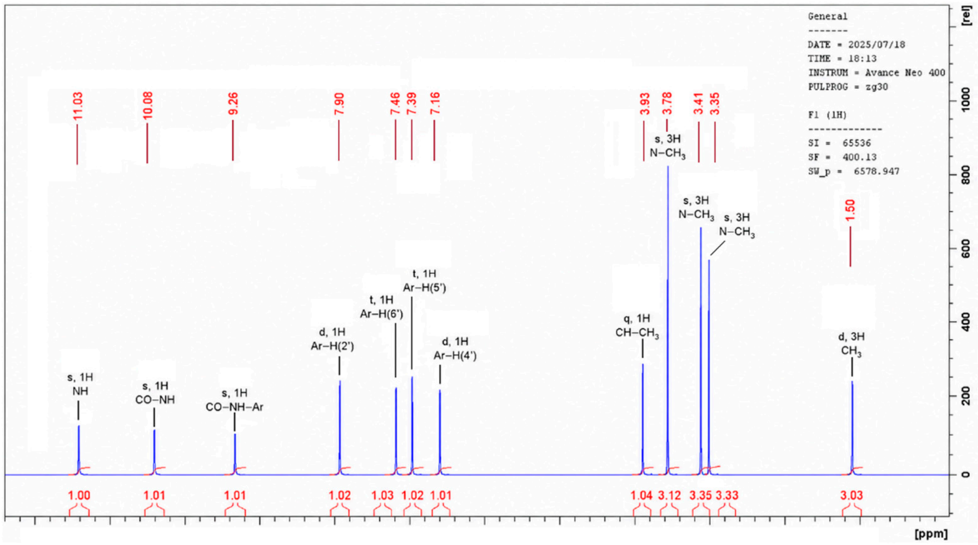

Supplementary Figure S3. ^{1}H NMR spectrum of JaS4.

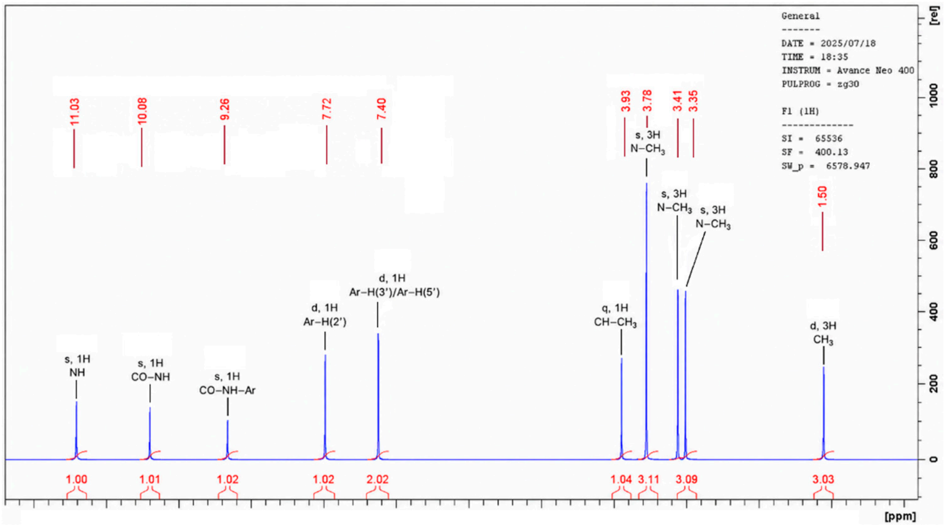

Supplementary Figure S4. ^{1}H NMR spectrum of JaS5.

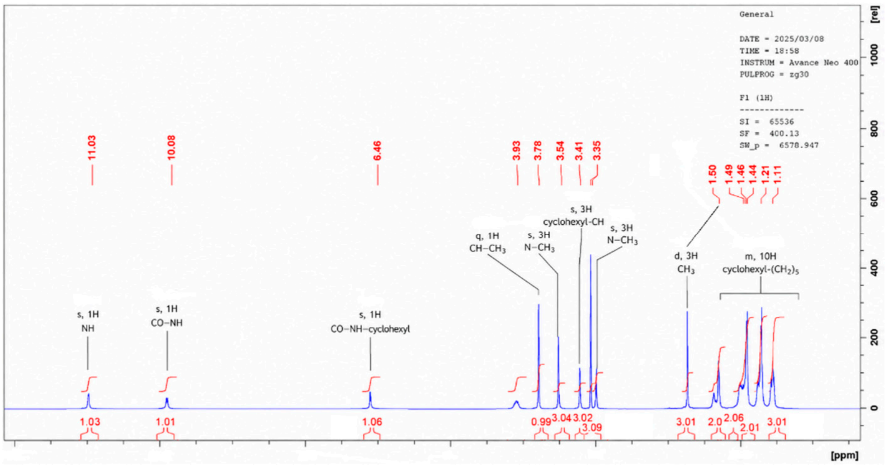

Supplementary Figure S5. ^{1}H NMR spectrum of JaS6.

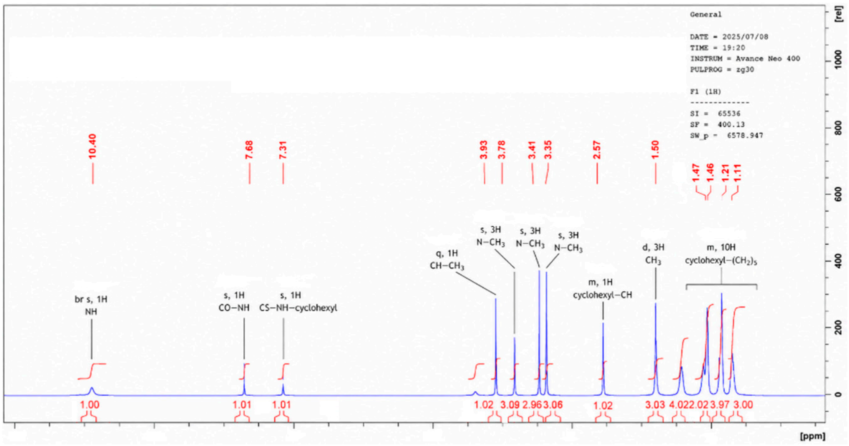

Supplementary Figure S6. ^{1}H NMR spectrum of JaS7.

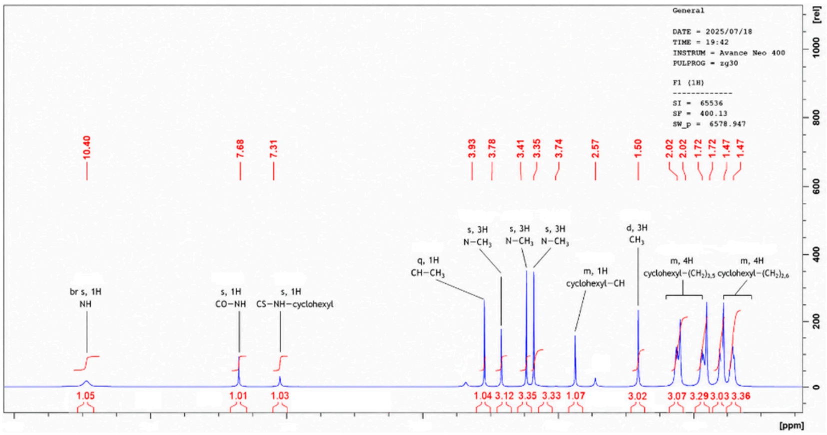

Supplementary Figure S7. ^{1}H NMR spectrum of JaS8.

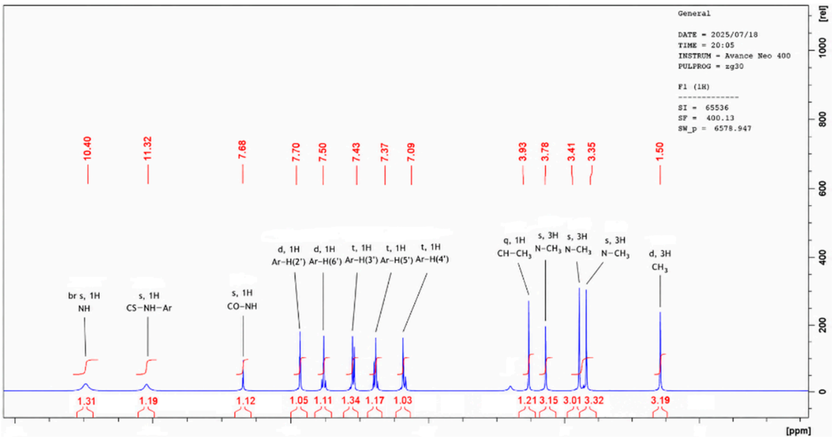

Supplementary Figure S8. ^{1}H NMR spectrum of JaS9.

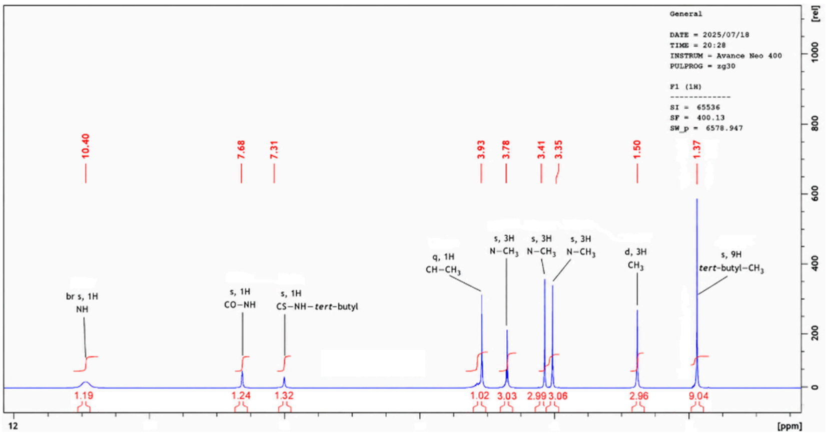

Supplementary Figure S9. ^{1}H NMR spectrum of JaS10.

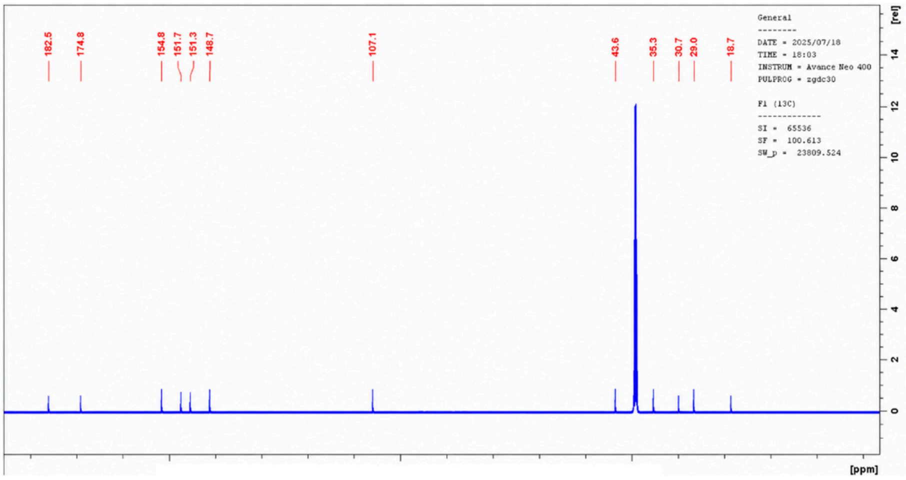

Supplementary Figure S10. ^{13}C NMR spectrum of JaS2.

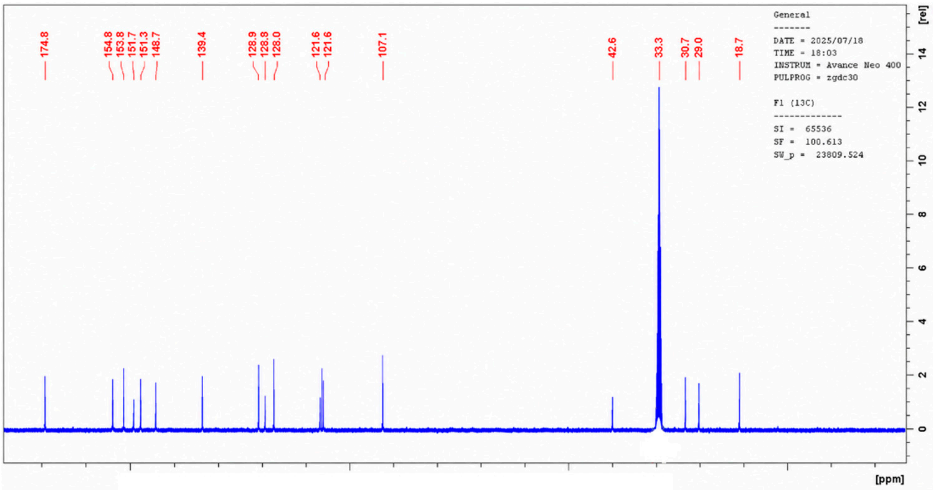

Supplementary Figure S11. ^{13}C NMR spectrum of JaS3.

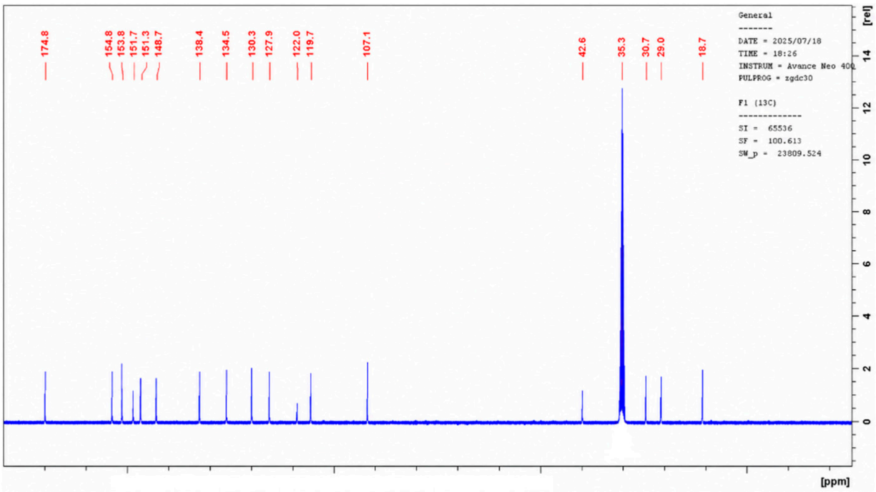

Supplementary Figure S12. ^{13}C NMR spectrum of JaS4.

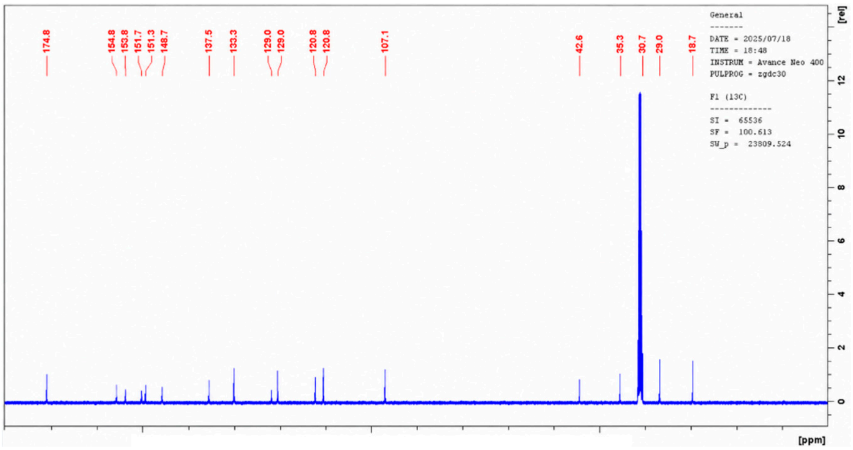

Supplementary Figure S13. ^{13}C NMR spectrum of JaS5.

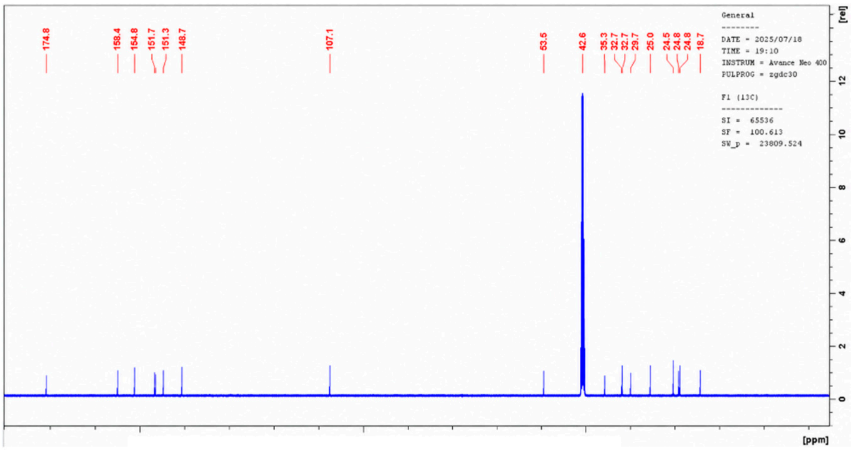

Supplementary Figure S14. ^{13}C NMR spectrum of JaS6.

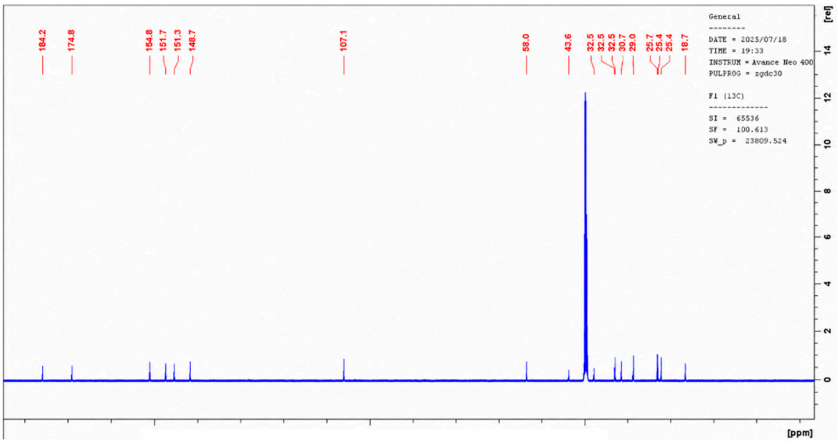

Supplementary Figure S15. ^{13}C NMR spectrum of JaS7.

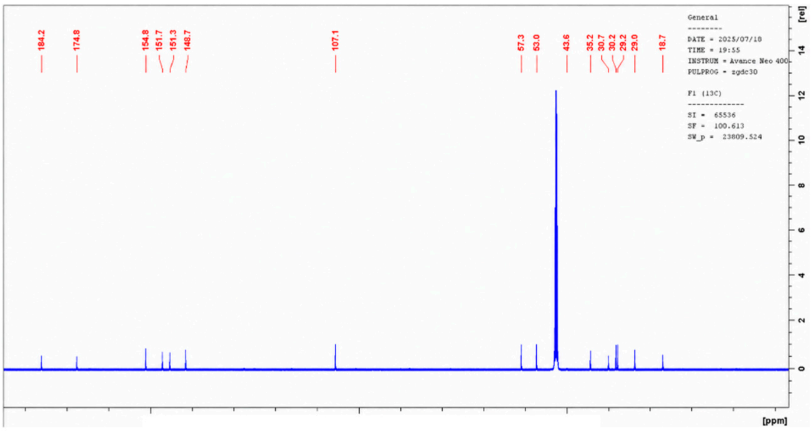

Supplementary Figure S16. ^{13}C NMR spectrum of JaS8.

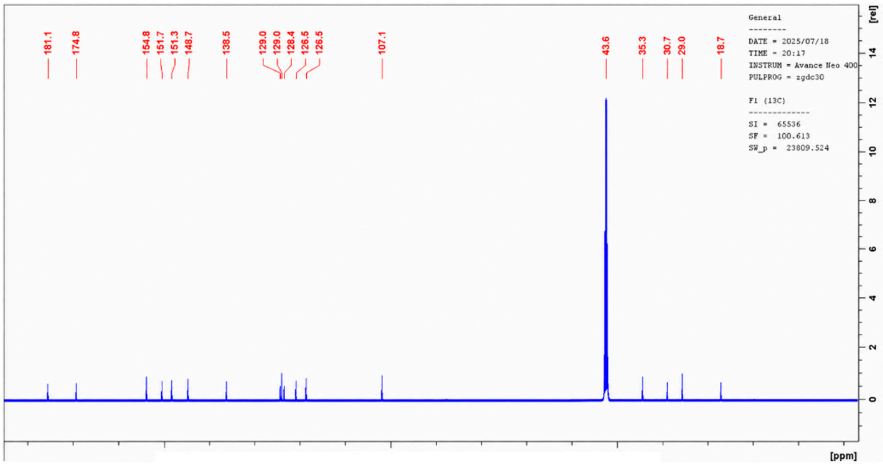

Supplementary Figure S17. ^{13}C NMR spectrum of JaS9.

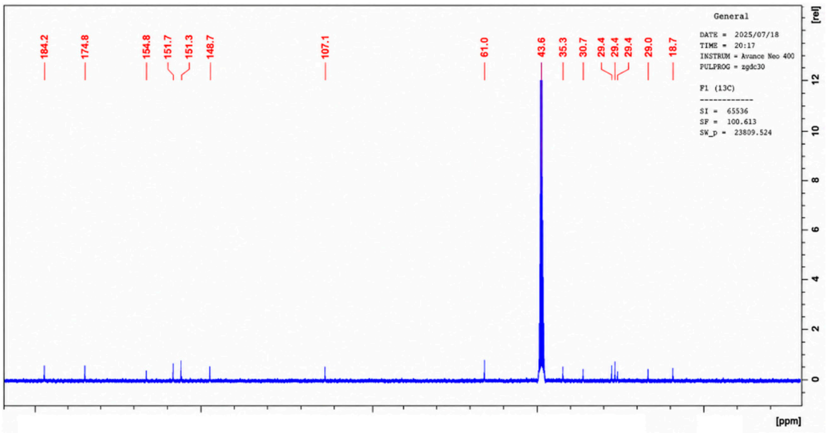

Supplementary Figure S18. ^{13}C NMR spectrum of JaS10.

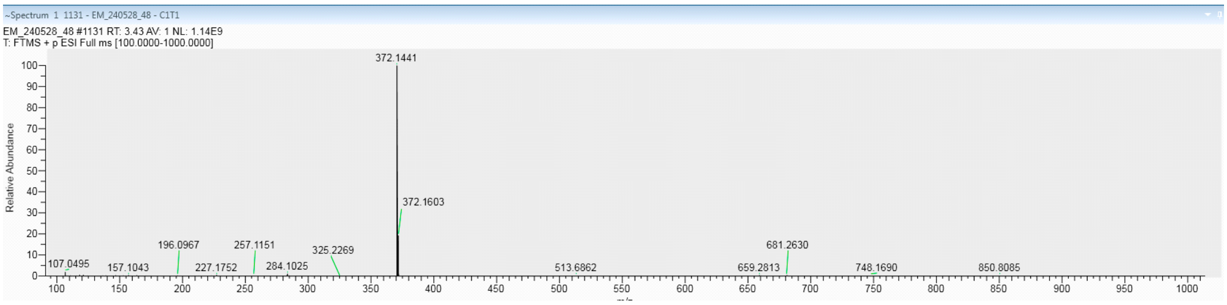

Supplementary Figure S19. LC-MS spectrum of JaS2.

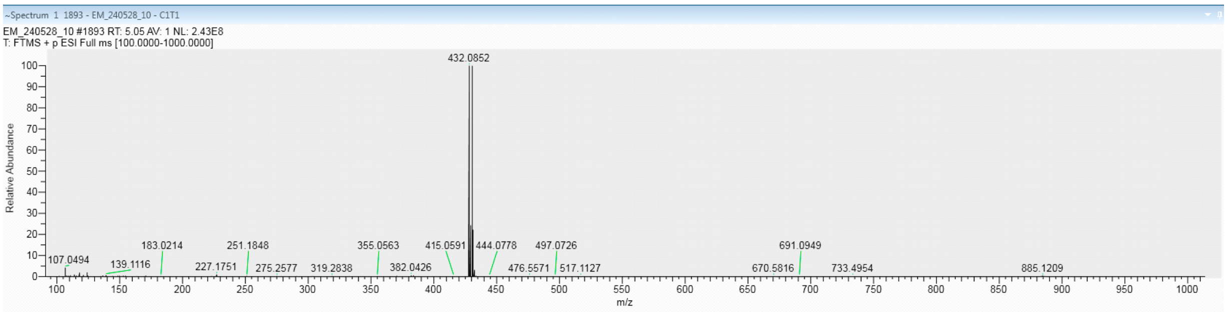

Supplementary Figure S20. LC-MS spectrum of JaS3.

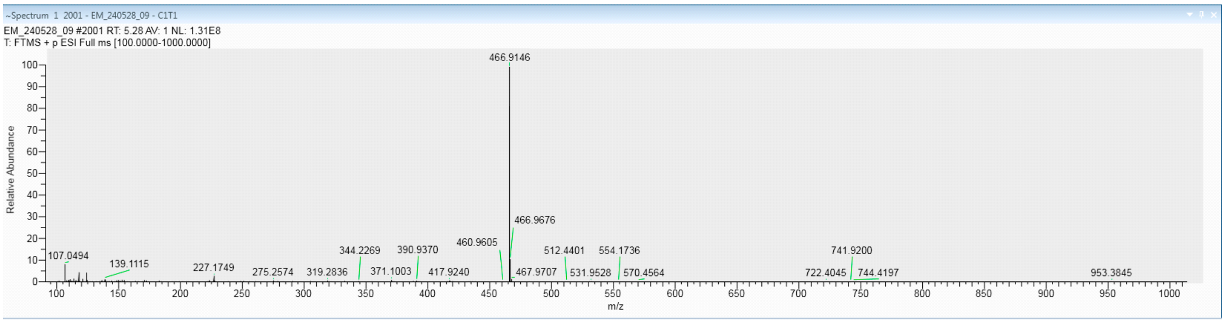

Supplementary Figure S21. LC-MS spectrum of JaS4.

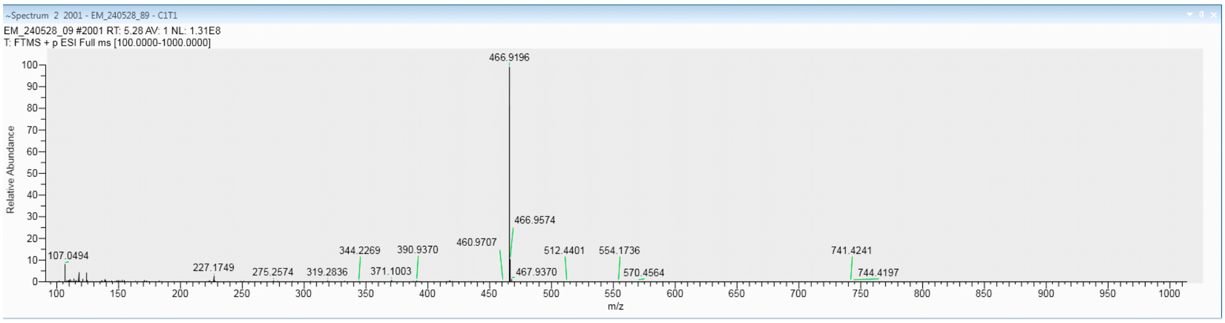

Supplementary Figure S22. LC-MS spectrum of JaS5.

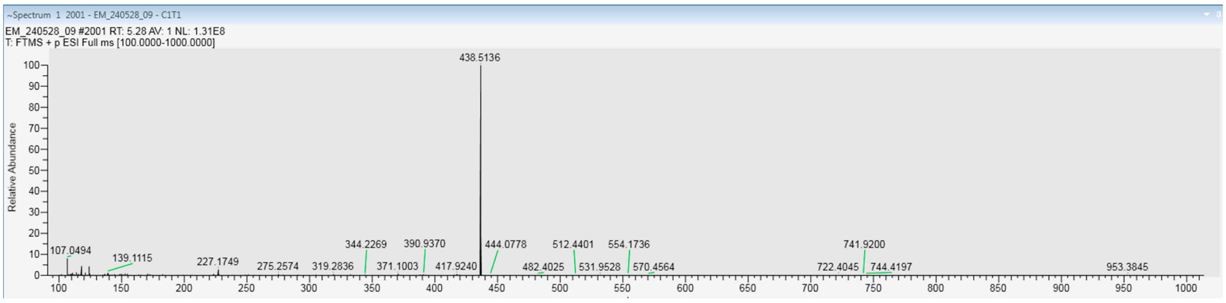

Supplementary Figure S23. LC-MS spectrum of JaS6.

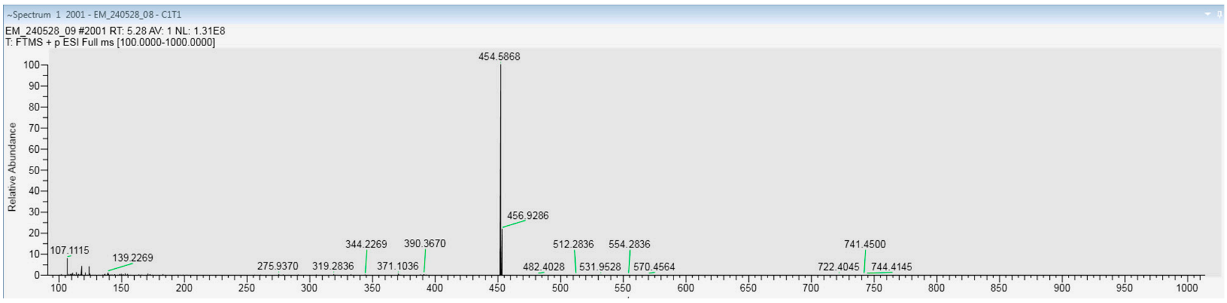

Supplementary Figure S24. LC-MS spectrum of JaS7.

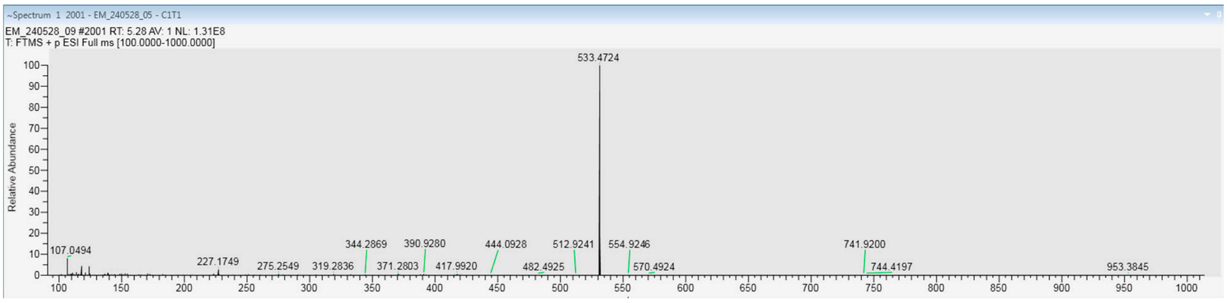

Supplementary Figure S25. LC-MS spectrum of JaS8.

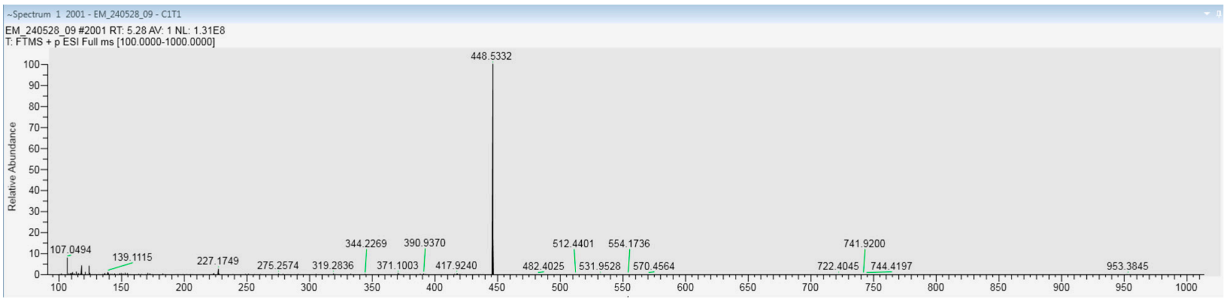

Supplementary Figure S26. LC-MS spectrum of JaS9.

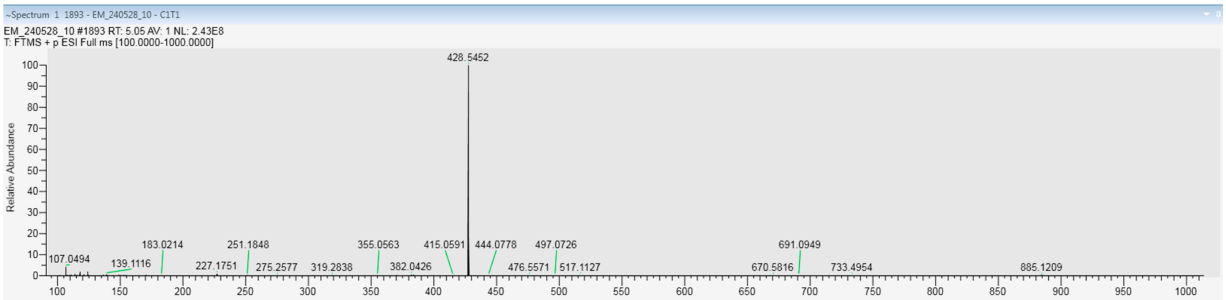

Supplementary Figure S27. LC-MS spectrum of JaS10.

Supplementary Table S1. Active monoamine oxidase B amino residues participating in the interactions with the top ranked xanthine derivatives and Safinamide.

Compound	Hydrophobic Interaction	Polar Interaction	Hydrogen Bond	π–π interaction	Steric clashes
**JaS7**	Tyr60, Pro102, Pro103, Pro104, Trp119, Leu164, Leu167, Phe168, Leu171, Cys172, Tyr188, Ile198, Ile199, Ile316, Leu328, Tyr326, Met341, Phe343, Tyr398, Tyr435	Ser200, Gln206	Ile199 (2.37Å), Cys172 (1.81Å), Tyr188 (1.76Å), water-mediated (1.74Å),	-	-
**JaS8**	Tyr60, Pro102, Pro103, Pro104, Trp119, Leu164, Leu167, Phe168, Leu171, Cys172, Tyr188, Ile198, Ile199, Ile316, Leu328, Tyr326, Met341, Phe343, Tyr398, Tyr435	His115, Gln206	Tyr188 (1.65Å)	Trp119 (4.11Å), Tyr398 (4.33Å)	-
**JaS9**	Tyr60, Pro102, Pro103, Pro104, Trp119, Leu164, Leu167, Phe168, Leu171, Cys172, Tyr188, Ile198, Ile199, Ile316, Leu328, Tyr326, Met341, Phe343, Tyr398	Ser200, Gln206	Tyr188 (2.05Å)	Tyr398 (4.34Å)	-
**JaS10**	Tyr60, Pro99, Pro102, Pro103, Pro104, Trp119, Leu164, Leu167, Phe168, Leu171, Cys172, Tyr188, Ile198, Ile199, Ile316, Leu328, Tyr326, Met341, Phe343, Tyr398, Tyr435	Ser200, Thr201, Gln206	-	Trp119 (4.11Å), Tyr398 (4.33Å)	-
**JaS5**	Tyr60, Pro102, Pro103, Pro104, Trp119, Leu164, Leu167, Phe168, Leu171, Cys172, Tyr188, Ile198, Ile199, Thr201, Gln206, Ile316, Tyr326, Leu328, Tyr326, Met341, Phe343, Tyr398, Gly434, Tyr435	Thr201, Gln206	Tyr188 (1.86Å)	Trp119 (4.16Å), Tyr398 (4.34Å)	-
**JaS3**	Tyr60, Pro102, Pro103, Pro104, Trp119, Leu164, Leu167, Phe168, Leu171, Cys172, Ser200, Tyr188, Ile198, Ile199, Ile316, Leu328, Tyr326, Met341, Phe343, Tyr398, Tyr435	Ser200, Gln206, Ser433	-	Tyr435 (3.92Å)	-
**JaS4**	Tyr60, Pro102, Pro103, Pro104, Trp119, Leu164, Leu167, Phe168, Leu171, Cys172, Ser200, Tyr188,	Gln206, Thr399	Tyr188 (1.99Å)	Trp119 (5.16Å), Tyr398 (4.32Å).	-

	Ile198, Ile199, Ile316, Leu328, Tyr326, Met341, Phe343, Tyr398, Tyr435				
**JaS6**	Tyr60, Pro99, Pro102, Pro103, Pro104, Trp119, Leu164, Leu167, Phe168, Leu171, Cys172, Tyr188, Ile198, Ile199, Ile316, Leu328, Tyr326, Met341, Phe343, Tyr398, Tyr435	Ser59, Gln206	Leu171 (2.27Å)	-	-
**JaS2**	Tyr60, Pro102, Pro103, Pro104, Trp119, Leu164, Leu167, Phe168, Leu171, Cys172, Tyr188, Ile198, Ile199, Ile316, Leu328, Tyr326, Met341, Phe343, Tyr398, Tyr435	Gln206	Cys172 (1.81Å), Tyr188 (1.76Å), Ile199 (1.98Å), H_2O (1.90Å),	-	-
**JaS1**	Tyr60, Trp119, Leu164, Leu167, Phe168, Leu171, Cys172, Tyr188, Ile198, Ile199, Ile316, Leu328, Tyr326, Met341, Phe343, Tyr398, Tyr435	Gln206	Cys172 (1.81Å), H_2O (1.93Å),	-	-
**Safinamide**	Tyr60, Phe103, Pro104, Leu171, Ile199, Gln206, Phe343, Tyr398, Tyr435	Gln206	Ile199, FAD	Leu171, Ile199, Ile316, Tyr326	-
